# Characterising access to healthcare and the health status of women domestic workers in Peru: a respondent-driven sampling study

**DOI:** 10.1136/bmjph-2025-004199

**Published:** 2026-02-05

**Authors:** Archna Gupta, Christopher Meaney, Karina M Romero, David Vera-Tudela, Maria Kathia Cardenas, María Sofía Cuba-Fuentes, Andrew D Pinto, Ayu Pinky Hapsari, Michael Anthony Rotondi, Janeth Tenorio-Mucha

**Affiliations:** 1 Upstream Lab, MAP Centre for Urban Health Solutions, Li Ka Shing Knowledge Institute, Unity Health Toronto, Toronto, Ontario, Canada; 2 Department of Family and Community Medicine, St Michael's Hospital, Toronto, Ontario, Canada; 3 Department of Family and Community Medicine, University of Toronto, Toronto, Ontario, Canada; 4 Universidad Peruana Cayetano Heredia, Lima, Peru; 5 CRONICAS Centre of Excellence in Chronic Diseases, Universidad Peruana Cayetano Heredia, Lima District, Peru; 6 Center for Research in Primary Health Care, Universidad Peruana Cayetano Heredia, Lima, Peru; 7 Dalla Lana School of Public Health, University of Toronto, Toronto, Ontario, Canada; 8 School of Kinesiology and Health Science, York University, Toronto, Ontario, Canada

**Keywords:** Health Services Accessibility, Sociodemographic Factors, Public Health

## Abstract

**Introduction:**

Domestic workers (DWs) worldwide face precarious and informal working conditions, including unstable employment, extended hours and insufficient labour protections, impacting their health. This study examines the health and healthcare access of women DWs in Peru, focusing on differences related to formal and informal employment.

**Methods:**

This participatory action research surveyed women DWs in Lima, La Libertad and Piura, Peru, from September 2023 to March 2024 using respondent-driven sampling (RDS). We gathered sociodemographic, healthcare access and health status data and reported counts and percentages with RDS-II estimators (95% CI). We compared health status and healthcare access by formal and informal employment using bootstrap RDS methods.

**Results:**

The study analysed data from 456 DWs. Most were between 19 and 45 years old (60.0%, 95% CI 50.1% to 70.0%), resided in Lima (69.8%, 95% CI 57.5% to 82.0%) and self-identified as Mestizo (54.8%, 95% CI 45.0% to 64.7%). Most respondents were precariously employed as informal DWs (90.5%, 95% CI 87.5% to 93.6%). A higher percentage of informal workers reported difficulty obtaining workplace permission for healthcare visits (32.2%, 95% CI 21.3% to 43.1% vs 21.2%, 95% CI 4.3% to 28.2%; p=0.187) and spent over 100 PEN (US$28) out of pocket on medical visits in the past year (21.2%, 95% CI 14.1% to 28.4% versus 10.5%, 95% CI 0.0% to 32.5%; p=0.249). Despite access to public health insurance, a higher percentage of informal workers visited private healthcare facilities (14.4%, 95% CI 8.3% to 20.5%) than formal workers (4.7%, 95% CI 1.5% to 7.8%) (p=0.084). A higher percentage of informal workers reported a diagnosis of depression (9.7%, 95% CI 5.1% to 14.4% vs 1.6%, 95% CI 0.0% to 4.4%; p=0.052) and anxiety (12.3%, 95% CI 6.1% to 18.5% vs 3.5%, 95% CI 0.0% to 9.1%; p=0.322). Obesity (43.0%) and depressive symptoms (41.4%) were prevalent across the DW population, regardless of employment status.

**Conclusions:**

Informal employment among women DWs in Peru may be linked to greater health challenges and barriers to care, despite public health insurance coverage. Addressing these inequities requires stronger labour protections.

**WHAT IS ALREADY KNOWN ON THIS TOPIC**

- Domestic workers, the majority of whom are women, face significant physical and mental health risks due to precarious and informal working conditions, limited legal protections and poor access to healthcare.

**WHAT THIS STUDY ADDS**

- This study is the first to comprehensively document the health status and healthcare access of women domestic workers in Peru, including formal and informal workers, using respondent-driven sampling. The study found high rates of obesity and depressive symptoms among all domestic workers. A higher percentage of informal workers faced financial and logistical barriers accessing care and often sought medical care from private health facilities or pharmacies.

**HOW THIS STUDY MIGHT AFFECT RESEARCH, PRACTICE OR POLICY**

- The findings underscore the need for stronger labour protections, expanded access to primary and mental healthcare, and more inclusive social protection for women domestic workers in Peru.

## Introduction

 The International Labour Organization (ILO) characterises domestic workers (DWs) as individuals employed in household(s) for various tasks such as cooking, cleaning, gardening, pet care, childcare, elderly care or driving family vehicles.^1^,^3^ Globally, there are an estimated 75.6 million people engaged in domestic work,^4^ with approximately 19.6% hailing from Latin America, where women make up the majority of this workforce.^5^ In this region, about one in every nine employed women works as a DW, many of whom are immigrants, indigenous peoples or Afro-descendants. These groups face a heightened risk of discrimination in the labour market, making domestic work one of their few available employment options.^5^

The health of DWs is shaped by the demanding and precarious working conditions. Globally, DWs are at elevated risk of both physical and mental health problems due to long hours, repetitive tasks and minimal rest or recovery time.^6^,^8^ These conditions are often accompanied by inadequate nutrition, exposure to harsh chemicals and limited access to medical care, resulting in higher rates of musculoskeletal pain, fatigue and chronic illnesses such as hypertension and diabetes.^6^,^8^ The mental health burden is similarly significant: DWs frequently report stress, anxiety and depression linked to job insecurity, isolation and, in some cases, verbal or physical abuse, particularly among live-in arrangements where personal and professional relationships are often blurred.^9 10^

The informality that characterises the domestic work sector is a major contributor to these vulnerabilities. In many countries, including Peru, DWs are often excluded from labour protections due to the absence of formal employment contracts or enrolment in social security systems.^11 12^ According to the ILO, over 75% of DWs globally are informally employed, lacking access to benefits such as paid leave, occupational health protections or health insurance.^13^ In Peru, although Law No. 31 047 was introduced in 2020 to formalise DW and extend labour rights to DWs, implementation remains limited.^14^ The ‘Domestic Workers Law’ (Law number 31047) approved in October 2020, establishes that (1) a workday and week consist of 8 hours and 48 hours, respectively; (2) payment of at least the minimum wage (around US$240); (3) mandatory healthcare insurance coverage through the Peruvian Social Security System (EsSalud) and maternity leave and (4) for DWs living in the workplace, provision of lodging and food by the employer in addition to the salary. However, 86.7% of DWs work under informal conditions and, therefore, do not benefit from the Domestic Worker Law given the absence of law enforcement. As a result, many DWs continue to experience exploitative working conditions and wage theft, which directly undermines their health and well-being and ability to access essential health and social services.^6 11^

Despite their economic and social contributions, DWs remain largely excluded from research and are rarely considered when creating social protection policies.^15^ This invisibility is particularly acute in Latin America, where the occupational, physical and mental health risks faced by DWs are rarely documented.^16^ If left unaddressed, these risks can lead to chronic and irreversible conditions that reduce productivity and force women DWs out of the economically active population, further deepening their risk of poverty. Furthermore, the unique experience of women DWs, shaped by intersecting identities such as migration status or indigenous languages as mother tongue, is seldom studied in the Peruvian context. The COVID-19 pandemic further exacerbated these inequities, as many DWs in Peru lost employment, benefits and access to adequate housing, while being excluded from most emergency social protection measures.^17^

Presently, there are limited reports on the socioeconomic characteristics and labour conditions of DWs.^17 18^ Even less is known about healthcare access and the health status of DWs. This study addresses these gaps by describing the health status and healthcare access of women DWs in Peru. Moreover, we sought to understand how their healthcare access and status vary according to their employment conditions, specifically whether DWs were formally or informally employed.

## Methods

### Study design

This study was part of a sequential mixed-methods study, titled ‘Addressing the challenges and constraints of social protection policies for Peruvian women domestic workers,” or the ANITA Project. The full protocol has previously been published.^19^ The overarching mixed-methods study was informed by the Social Determinants of Health (SDOH) Framework established by the WHO and the Gender Equity and Social Inequality Framework.^20 21^ The SDOH framework examines the complex interactions among social, economic and political factors that influence health outcomes. Moss’s framework further incorporates considerations such as the geopolitical context, cultural norms and sanctions, women’s societal roles, mediating health factors and resultant health outcomes.

In phase 2 of this study, we conducted face-to-face surveys to expand on secondary data analyses to better understand the working conditions, health conditions and healthcare access of Peruvian women DWs.^22^

### Public and patient involvement

This study used a participatory action research approach, where women DWs served as coresearchers, involved in designing the survey, recruiting participants and validating the results.^23^ Comprehensive details regarding participant involvement are provided in our published protocol.^19^

### Setting and context

The data were collected in three different cities in Peru with a high concentration of DWs according to the National Household Survey (ENAHO 2020)^24^: Lima (the capital), La Libertad and Piura (located on the Northern coast of Peru).

This survey was conducted between September 2023 and December 2024 in Lima, and between February 2024 and March 2024 in La Libertad and Piura. This survey took place following the COVID-19 pandemic. We believe that DWs’ working conditions may have been at their worst during the pandemic. Related research found that female DWs in Peru experience persistent health disadvantages before, during and after the COVID-19 pandemic when compared with females with formal employment.^22^ Given the paucity of literature on DW health status and access to health, estimates of health outcomes and access to health are relevant for decision-makers.

### Participants and recruitment

The study population was women DWs. The ILO defines a DW as a *“worker who performs work in or for a private household or households. They provide direct and indirect care services, and as such are key members of the care economy. Their work may include tasks such as cleaning the house, cooking, washing, and ironing clothes, taking care of children, or elderly or sick members of a family, gardening, guarding the house, driving for the family, and even taking care of household pets”*.^25^

We included individuals older than 14 years of age, who self-identified as women and were working as DWs as their primary source of income. The age cut-off of 14 aligns with the National Household Survey (ENAHO, 2021).^24^ DWs could be working on a full-time or part-time basis, employed by a single or multiple households or through a service provider, and residing in the household of the employer (live-in worker) or living in her own residence (live-out). The participants must have been residing in one of the study communities (Lima, La Libertad, Piura) for at least 6 months at the time of enrolment. We excluded individuals doing unpaid DW or where DW was not their primary source of income, or those who could not provide informed consent.

We used respondent-driven sampling (RDS), a network-based sampling method that begins with a small convenience sample (known as ‘seeds’) and that incentivises respondents to participate in the survey and refer their peers.^26^ Using peer referrals, RDS can reach vulnerable groups, such as the DWs in Peru, who may face repercussions for participating in a research study. RDS assumes that the population is socially connected, as recruitment into the study is based on their peers.

RDS differs from traditional snowball sampling in two ways: first, it involves a dual incentive system, including a reward for participating and a reward for recruiting others into the study; second, initial subjects are not asked to identify their peers to the investigator but to recruit them to the study themselves. The advantage of RDS over snowball sampling is its ability to generate asymptotically unbiased estimates of population characteristics by accounting for participants’ network size and recruitment patterns.^27 28^ RDS studies also collect additional information about respondents, including the number of connections they have in the population of interest and information on the recruitment chains to enable statistical adjustments to the observed proportions.^29^

To promote a sample that is representative of the DWs’ population in the three cities, DW coresearchers and the study team purposely recruited seeds with diverse ages, DW area of residence, employer’s area of residence and division of labour (full-time vs part-time or live-in vs live-out). We recruited 12 seeds in Lima, 4 in La Libertad and 5 in Piura. Each participant was limited to three peer referrals (although one participant recruited four); this limit was intended to encourage equal referral opportunities across all participants. The research team closely monitored the chain’s expansion, gradually reducing the number of referrals to two, one and eventually zero near study completion.

Participants received a dual incentive: 70 PEN (~US$20) on survey completion and 35 PEN (~US$10) for each referred participant who consented to participate. Each coupon (online supplemental file 1) included a phone number for referrals to contact recruiters. Recruiters verified the coupon code, conducted a prescreening phone call to confirm eligibility and scheduled data collection. In Lima, participants could choose between two university offices or the Union’s offices. In La Libertad and Piura, data were collected at a single location per region.

### Data sources and collection

We collected data on DW sociodemographic characteristics, healthcare access and health status (online supplemental file 2). The survey was developed iteratively, with the DW coresearchers testing different survey versions and providing input on the relevance of questions to current issues, the clarity of the questions and the overall user experience of the survey. The survey consisted of 259 questions and took approximately 60 min to complete.

Given the study population and the sensitivity of some questions, all research team members who came into contact with participants were trained in research ethics, gender-sensitive data collection, psychological first aid and study procedures. Data collection was performed by women using tablets and managed in Research Electronic Data Capture hosted at Universidad Peruana Cayetano Heredia in Peru.^30^ After participation, all respondents received printed materials on the Domestic Workers Law, written contract templates and contact information for support services in case they had concerns about violence at home or work.

### Outcomes

#### Sociodemographic characteristics

Sociodemographic data included age, interview location, gender identity, marital status, migration status, level of education, self-identified ethnicity, mother tongue, whether they were the head of the household, whether they were the main contributor to household income, and, if they were living with children, how many children they had. Socioeconomic status was assessed by asking about access to basic services, including electricity and water. We also collected information about whether participants belonged to a DW organisation including unions, training groups and advocacy groups, if they were employed formally (registered contract with the ministry of labour, notarised employment contract, written employment contract, contract with outsourcing agency) or informally (verbal agreement only or no contract or agreement), and how many years they had been working as a DW.

#### Healthcare access

To assess healthcare access, we asked about the type of health insurance they had, if any. The Ministry of Health (MINSA, or Ministerio de Salud, in Spanish), through the Integral Health Insurance (SIS or Seguro Integral de Salud, in Spanish), targets the poor and the extremely poor groups. The Social Security (EsSalud or Seguro Social de Salud del Peru, in Spanish) provides formal insurance to employees and their beneficiaries. The Armed Forces and the National Police Medical Services both provide insurance to their workers’ direct family, including children and spouses. Private sector institutions offer insurance to those who can afford to pay their premiums.^31^ We also collected information on whether DWs contribute to private health insurance and whether their employers pay for EsSalud health insurance.

We also assessed the type of facility from which they accessed care in the past twelve months and the type of care they received (primary care vs emergency department). For the type of facility, they were classified as public (MINSA primary care centre or hospital, EsSalud primary care centre or hospital, or police or armed forces hospital), private (private clinic, private office, solidarity hospital, municipal service and non-governmental organisation or religious organisation service) or a pharmacy. We also assessed DW’s perceived barriers to care, including difficulties in finding medical care and trouble obtaining permission to leave work. We also asked about out-of-pocket expenses for medical care. Finally, if patients had been involved in an accident at work, we asked whether their employer had provided medical services.

#### Health status

We asked about perceived health status and diagnosed health conditions. We also asked about depressive symptoms using the Centre for Epidemiologic Studies Depression Scale (CES-D),^32^ a 10-item self-report measure of the occurrence of depressive symptoms ‘during the past week’ using a 5-point Likert scale. A score of 10 or more is considered screen positive for depressive symptoms. We also specifically asked about health conditions related to the participants’ work as DWs. Finally, we asked about COVID-19 infection and vaccination.

The questions asked are available in online supplemental file 2 in Spanish, the language in which the surveys were conducted.

### Sample size

The sample size was calculated based on average working hours per week, given that the primary outcome of this overall cross-sectional study was understanding the working hours per week and the hourly wage of DWs.^19^ The sample size was estimated on the National Household Survey’s (ENAHO-2021) reporting that women who self-identified as DW reported working an average of 40.4 hours per week (SD: 19.1 hours per week).^24^ Given the variability about DW self-reported working hours, we estimated that a sample of size N=224 would be required to make 95% confidence statements with a precision (ie, CI total width) of 5 hours. Previous work with RDS designs suggests that design effects can be large, and sample size inflation is necessary to maintain power and type 1 error rates at prespecified levels.^33^ Balancing time, financial and human resource constraints, we assumed an RDS design effect of two, resulting in a final sample size of N=448.

The sample size (N=448) relates to the design of a study aiming to estimate a single continuous endpoint (ie, hours worked) with a given precision. This study leverages the data collected for the overarching ANITA project as described in the study design section. It was not designed a priori to detect potentially meaningful differences in healthcare access and health status between formally and informally employed DWs, as specified in the overarching sample size plan.

### Statistical analysis

We summarise categorical variables using counts and percentages. We estimated 95% CIs for all variables using the RDS package (V.0.9.2) in R statistical software (V.4.0.2) with the RDS-II estimator.^34 35^ This estimator assigned a weight to each participant based on their sampling probability, as estimated using reported network size. The survey measured the network size of each participant by asking: “How many domestic workers do you know in the region where you live, and how many domestic workers have you communicated with in the last year, and how many domestic workers have you communicated with in the last week?”

We also investigated differences in health status and access to healthcare across formal and informal employment statuses using bootstrap RDS methods. In particular, we used the RDS::bootstrap.contingency.table() function from the RDS package—with RDS-II weights, B=10 000 bootstrap replicate samples and a single randomly chosen fixed seed for reproducibility.^36^ P values were inestimable if the underlying contingency table contained zero-valued cells. Statistical significance was declared if the observed bootstrap p value was less than 0.05.

## Results

We assessed 489 individuals for study eligibility and excluded 33 during the prescreening phone call. Reasons for exclusion included occasional DW (n=14), working as janitors or cleaners (n=12), DW as a secondary source of income (n=2) and unemployment (n=5). We interviewed and analysed data from a total of 456 DWs, between the ages of 15 and 77, which included 21 seeds and 435 recruited participants.

**Figure 1:**
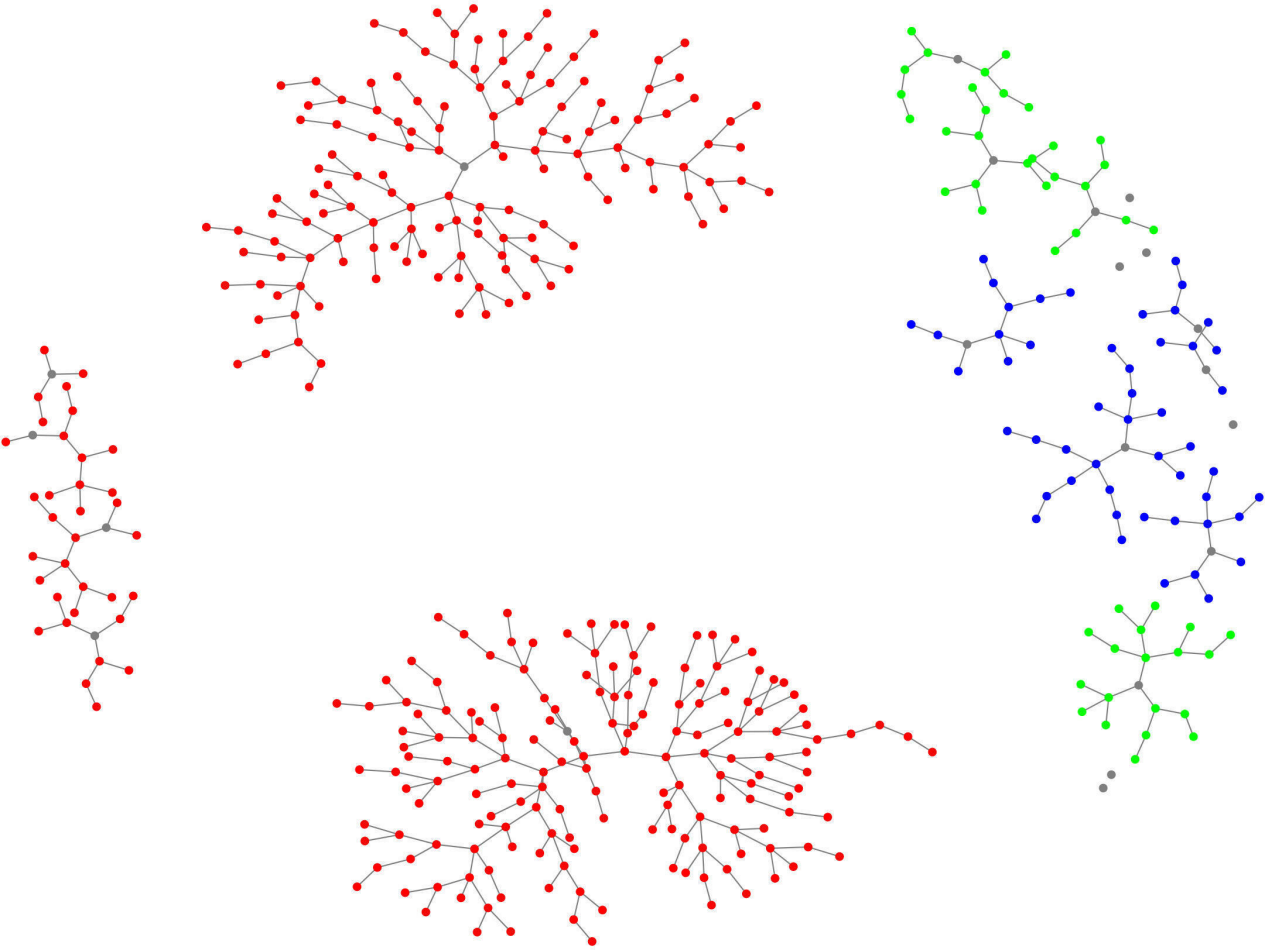
Network diagram illustrating recruitment and connectedness of DWs included in the ANITA phase 2 study sample. Red dots represent recruited participants from Lima, blue from Piura and green from La Libertad, while grey dots represent seeds. DWs, domestic workers.

Figure 1 presents the RDS recruitment diagram, showing waves propagating from each seed, as well as the number of recruits per seed. The maximum number of recruitment waves for any given chain was 12.

Participant demographic characteristics are described in table 1. Most respondents lived and worked in Lima (n=350), followed by Piura (n=55), then La Libertad (n=51). Nearly all respondents self-identified as cisgender females. Most respondents were between the ages of 19 and 45 (n=265), had a secondary education or higher (n=232) and self-identified as mestizo (people of mixed entities) (n=233). 102 women identified as internal migrants, while 16 identified as international migrants.

**Table 1:** Sociodemographic characteristics of domestic workers

Characteristic	Unweighted no. of participants	Unadjusted estimate, %	RDS-II adjusted estimate, % (95% CI)
Age (n=456)			
14–18	13	2.9	4.3 (1.1 to 7.4)
19–45	265	58.1	60.0 (50.1 to 70.0)
46–64	165	36.2	32.7 (22.8 to 42.7)
65+	13	2.9	3.0 (0.0 to 6.0)
Location (n=456)			
Lima	350	76.8	69.8 (57.5 to 82.0)
La Libertad	51	11.2	17.0 (4.3 to 29.8)
Piura	55	12.1	13.2 (5.6 to 20.8)
Marital status (n=456)			
Partnered	187	41.0	38.4 (29.6 to 47.1)
Other	269	59.0	61.6 (52.9 to 70.4)
Migration status (n=444)			
Non-migrant	326	73.4	72.7 (61.4 to 83.9)
Internal migrant	102	23.0	21.3 (14.0 to 28.7)
International migrant	16	3.6	6.0 (0.0 to 17.3)
Level of education (n=364)			
Less than secondary school	132	36.3	34.6 (22.5 to 46.6)
Secondary school or greater	232	63.7	65.4 (53.4 to 77.5)
Self-reported ethnicity (n=456)			
Afro-Peruvian or Afrodescendant	43	9.4	7.7 (4.7 to 10.7)
Indigenous	86	18.9	15.1 (5.3 to 24.9)
Mestizo	233	51.1	54.8 (45.0 to 64.7)
Other	94	20.6	22.3 (14.8 to 29.9)
Language/mother tongue (n=456)			
Spanish	432	94.7	95.6 (93.9 to 97.2)
Indigenous	24	5.3	4.4 (2.8 to 6.1)
Access to essential services: electricity (n=456)			
Public network with own metre	269	59.0	62.3 (53.4 to 71.2)
Network with shared metre	161	35.3	33.0 (24.5 to 41.5)
Other	26	5.7	4.7 (1.6 to 7.7)
Access to essential services: water (n=456)			
Public network with own metre	224	49.1	51.4 (41.5 to 61.4)
Public network, with shared metre	170	37.3	35.1 (26.0 to 44.3)
Other	62	13.6	13.4 (7.5 to 19.4)
Head of Household (n=456)			
Yes	226	49.6	43.9 (33.8 to 54.0)
Primary contributor to household income (n=455)			
No or all equal	227	49.9	54.8 (44.5 to 65.0)
Yes	228	50.1	45.2 (35.0 to 55.5)
Lives alone (n=456)			
Yes	28	6.1	5.9 (0.6 to 11.2)
# children living with DW (n=343)			
1	127	37.0	40.7 (29.5 to 51.9)
2	118	34.4	32.6 (23.3 to 41.8)
3+	98	28.6	26.7 (17.8 to 35.6)
Affiliated with a DW organisation (n=456)			
Yes	43	9.4	7.5 (3.9 to 11.1)
Employment status (n=456)			
Formal	49	10.7	9.5 (6.4 to 12.5)
Informal	407	89.3	90.5 (87.5 to 93.6)
Work experience (n=441)			
Less than 1 year	11	2.5	3.5 (0.0 to 7.8)
1–5 years	173	39.2	48.2 (37.8 to 58.7)
> 5 years	257	58.3	48.3 (37.6 to 58.9)

DW, domestic worker; RDS, respondent-driven sampling.

The vast majority (n=407) were informal DWs. Similar percentages indicated working for 1–5 years (N=173) and greater than 5 years (n=257), with a few working less than 1 year (n=11).

Data on DWs’ self-reported healthcare access are presented in table 2. Corresponding p values are provided in online supplemental file 3, which contains the full version of table 2. Most respondents (72.6%) have healthcare coverage through the public Integral Health Insurance (SIS) programme. However, when we stratified respondents by formal or informal work type, we found that a greater percentage of formal workers used EsSalud (28.2%, 95% CI 6.5% to 49.8%) than informal workers (14.8%, 95% CI 7.6% to 22.0%) (p=0.129). Relatedly, we found that employers were more likely to pay for EsSalud for formal workers than informal workers (p=0.005). We asked participants why they were not affiliated with the EsSalud system. 148 (39.3%) indicated they preferred a different health insurance, 65 (17.2%) indicated their employer opposed affiliation, 29 (7.7%) indicated they worked part-time or had multiple employers, 16 (4.2%) preferred cash contribution over insurance and 9 (2.4%) felt they did not need health insurance.

**Table 2:** DW access to healthcare stratified by formal versus informal employment contract status

Characteristic	Overall		Formal			Informal		
	Unadjusted		RDS-II adjusted estimate			RDS-II adjusted estimate		
	N	%	N	%	95% CI	N	%	95% CI
Type of health Insurance	456		49			407		
Integral health insurance (SIS)	331	72.6	29	57.7	(35.4 to 79.9)	302	73.0	(61.6 to 84.4)
Social security insurance (EsSalud)	78	17.1	14	28.2	(6.5 to 49.8)	64	14.8	(7.6 to 22.0)
Private insurance	47	10.3	6	14.2	(1.4 to 27.0)	41	12.2	(1.1 to 23.4)
DW contributes to health insurance	409		43			366		
Yes	26	6.4	9	18.1	(3.0 to 33.3)	17	5.2	(0.0 to 12.4)
Employer paid for EsSalud insurance	76		14			62		
Yes	23	30.3	10	64.8	(19.0 to 100.0)	13	23.4	(0.0 to 48.2)
Type of facility accessed in past 12 months								
Public	456		49			407		
Yes	393	86.2	46	93.6	(70.8 to 100.0)	347	83.8	(72.9 to 94.8)
Private	456		49			407		
Yes	65	14.3	4	4.7	(1.5 to 7.8)	61	14.4	(8.3 to 20.5)
Pharmacy	456		49			407		
Yes	31	6.8	2	6.0	(0.0 to 29.2)	29	7.7	(0.0 to 18.5)
Healthcare type								
Primary healthcare provider	453		49			404		
Yes	161	35.5	10	14.4	(2.0 to 26.9)	151	36.3	(27.6 to 45.0)
Emergency department	456		49			407		
Yes	121	26.5	14	30.2	(13.9 to 46.4)	107	24.0	(16.4 to 31.5)
Trouble finding medical care	456		49			407		
Yes	173	37.9	17	33.8	(12.0 to 55.6)	156	36.5	(27.2 to 45.9)
Trouble getting workplace permission for HC visit	453		49			404		
Yes	151	33.3	13	21.2	(4.3 to 38.2)	138	32.2	(21.3 to 43.1)
Out-of-pocket expense of last medical visit in past 12 months	440		48			392		
Greater than 100 PEN	99	22.5	6	10.5	(0.0 to 32.5)	93	21.2	(14.1,28.4)
100 PEN or less	143	32.5	15	33.0	(15.5 to 50.6)	128	34.2	(22.5,45.9)
Nothing to everything is covered by insurance	198	45.0	27	56.4	(33.6 to 79.3)	171	44.5	(34.1,54.9)
Accident at work in the last 12 months	456		49			407		
Yes	62	13.6	10	20.6	(0.0 to 43.0)	52	11.6	(4.1 to 19.1)
Medical services provided by the employer?	62		10			52		
Yes	25	40.3	*	11.0	(0.0 to 36.2)	*	46.8	(20.2 to 73.4)
No	15	24.2	*	29.4	(0.0 to 85.9)	*	19.8	(3.6 to 35.9)
Medical attention not needed	22	35.5	5	59.7	(17.8 to 100.0)	17	33.5	(6.7 to 60.2)

For corresponding p values, please see the full version of table 2 in online supplemental file 3.

* Data were suppressed for cells with fewer than five individuals to protect individual confidentiality and prevent the calculation of sensitive data through subtraction from corresponding totals.

DW, domestic worker; RDS, respondent-driven sampling.

Interestingly, a higher percentage of informal workers accessed private healthcare facilities (14.4%, 95% CI 8.3% to 20.5%) than formal workers (4.7%, 95% CI 1.5% to 7.8%) (p=0.084). However, both formal and informal workers most often used public health facilities (86.2%). We also asked participants if they had a ‘primary healthcare provider’ they visited most frequently. Informal workers were more likely to answer ‘yes’ to this question (36.3%, 95% CI 27.6% to 45.0%) compared with formal workers (14.4%, 95% CI 2.0% to 26.9%)(p=0.017). If any respondent answered ‘yes’, they were asked to specify the type of provider they had seen. In total, 80 participants provided details. Of this sample, 18% identified a general practitioner or family physician, 60% indicated a specialist (including cardiologist, dermatologist, endocrinologist, gynaecologist, obstetrician, gastroenterologist, haematologist, psychiatrist, ophthalmologist and rheumatologist), and 18% indicated a non-physician health worker (including a nurse, pharmacist, dentist or psychologist). Interestingly, we found that formal workers (30.2%, 95% CI 13.9% to 46.4%) were more likely than informal workers (24.0%, 95% CI 16.4% to 31.5%) to have visited an emergency department in the past 12 months (p=0.486).

We also sought to understand DW perceptions of barriers to care. A higher percentage of informal workers (32.2%, 95% CI 21.3% to 43.1%) reported having trouble getting workplace permission to visit a health facility when asked, “When you get sick and require medical treatment, is it a problem for you to get permission from your workplace to go for treatment?” compared with formal workers (21.2%, 95% CI 4.3% to 38.2%) (p=0.187). Similarly, more informal workers (21.1%, 95% CI 14.1% to 28.4%) spent greater than 100 PEN (US$28) on medical visits in the past 12 months than formal workers (10.5%, 95% CI 0.0% to 32.5%) (p=0.249). However, more formal workers reported experiencing a workplace accident (20.6%, 95% CI 0.0% to 43.0%) than informal workers (11.6%, 95% CI 4.1% to 19.1%) (0.167).

In table 3, we provide additional information on the challenges DWs face when accessing healthcare.

**Table 3:** Domestic worker-reported challenges when accessing healthcare

Response	Count	Unadjusted per cent (%)
It is difficult due to medication shortages.	413	91.0
It is difficult because the dates for medical appointments are far away.	385	84.4
It is difficult because of the paperwork (lack of an integrated health system, which requires repetitive paperwork at every point of entry)	370	81.1
It is difficult because there are no health personnel.	342	75.2
It is difficult to get money for transportation, consultation and/or treatment.	198	43.4
It is difficult that there are no female health workers.	192	42.1
I do not know where to go for treatment.	173	37.9
It is difficult to get permission from your workplace to go for treatment.	151	33.3
The distance to medical services is a challenge.	144	31.7
It is difficult to get transport to the health facility.	129	28.3
It is difficult to go to the health facility alone.	116	25.4

Results regarding the self-reported health status of DW respondents are available in table 4. Corresponding p values are provided in online supplemental file 4, which contains the full version of table 4. Higher percentages of formal workers self-reported diagnoses of hypertension, high cholesterol, back pain and sleep disorders. Higher percentages of informal workers reported diabetes, asthma, depression and anxiety. Large percentages of both informal and formal workers reported being overweight (body mass index, BMI 25–30 kg/m^2^) and obese (BMI <30 kg/m^2^).

**Table 4:** DW health status stratified by formal versus informal employment contract status

Characteristic	Overall		Formal			Informal		
	Unadjusted		RDS-II adjusted estimate			RDS-II adjusted estimate		
	N	%	N	%	95% CI	N	%	95% CI
Perceived health status	456		49			407		
Excellent and very good	46	10.1	4	8.2	(0.0 to 28.3)	42	11.6	(5.1 to 18.2)
Good to fair and bad	410	89.1	45	91.8	(71.7 to 100.0)	365	88.4	(81.8 to 94.9)
Hypertension	452		49			403		
Yes	34	7.5	*	8.9	(1.1 to 16.7)	*	8.2	(2.9 to 13.6)
Diabetes	433		49			406		
Yes	22	4.8	*	4.4	(0.0 to 27.9)	*	5.1	(0.8 to 9.4)
Hypercholesterolaemia	454		49			405		
Yes	75	16.5	12	27.0	(2.9 to 51.2)	63	13.9	(8.3 to 19.5)
Asthma	456		49			405		
Yes	22	4.8	*	1.6	(0.0 to 3.3)	*	4.3	(1.1 to 7.5)
Back pain	456		49			407		
Yes	25	5.5	*	11.2	(0.0 to 35.7)	*	5.1	(2.3 to 7.9)
Depression	455		49			406		
Yes	40	8.8	*	1.6	(0.0 to 4.4)	*	9.7	(5.1 to 14.4)
Anxiety	454		49			407		
Yes	48	10.5	*	3.5	(0.0 to 9.1)	*	12.3	(6.1 to 18.5)
Sleep disorder	456		49			407		
Yes	24	5.3	*	10.3	(0.0 to 35.9)	*	5.8	(1.7 to 10.0)
BMI	456		49			407		
<25.0	95	20.8	11	24.1	(7.4 to 40.9)	84	23.6	(14.3 to 32.8)
25.0 to <30.0	165	36.2	16	38.9	(14.3 to 63.6)	149	36.4	(27.2 to 45.7)
30.0+	196	43.0	22	36.9	(19.6 to 54.3)	174	40.0	(29.3 to 50.8)
Depression based on CES-D scale	456		49			407		
Yes	189	41.4	19	34.3	(12.6 to 55.9)	170	41.7	(31.9 to 51.4)
Disability condition	456		49			407		
Yes	9	2.0	0	0	†	9	1.5	(0.4 to 2.5)
Any illness	456		49			407		
Yes	39	8.6	*	1.4	(0.0 to 3.0)	*	6.8	(2.5 to 11.2)
Skin disease	456		49			407		
Yes	5	1.1	*	0.6	(0.0 to 1.1)	*	0.7	(0.3 to 1.1)
MSK disease	456		49			407		
Yes	29	6.4	*	1.4	(0.0 to 3.0)	*	5.3	(1.1,9.5)
COVID-19 infection	456		49			407		
No or do not remember	257	56.4	31	67.8	(45.4 to 90.3)	226	57.3	(46.7 to 67.9)
Yes	199	43.6	18	32.2	(9.7 to 54.6)	181	42.7	(32.1 to 53.3)
COVID-19 vaccine	456		49			407		
1 dose	8	1.8	0	0	†	8	1.1	(0.2 to 1.9)
2 doses	40	8.8	0	0	†	40	10.6	(4.1 to 17.1)
3 or more doses	402	88.2	49	100	†	353	86.9	(80.2 to 93.6)
No doses	6	1.3	0	0	†	6	1.5	(0.0 to 3.1)

For corresponding p values, please see the full version of table 4 in online supplemental file 4.

* Data were suppressed for cells with fewer than five individuals to protect individual confidentiality and prevent the calculation of sensitive data through subtraction from corresponding totals.

† RDS-II CI not estimable—since the point estimate of the percentage is 0% or 100%.

CES-D, Centre for Epidemiologic Studies Depression; DW, domestic worker; RDS, respondent-driven sampling.

Few DWs indicated a diagnosis of depression; however, a larger percentage of informal workers (9.7%, 95% CI 5.1% to 14.4%) reported this diagnosis when compared with formal workers (1.6%, 95% CI 0.0% to 4.4%) (p=0.052). Large proportions of informal (41.7%, 95% CI 31.9% to 51.4%) and formal workers (34.3%, 95% CI 12.6% to 55.9%) screened positive for depressive symptoms when using the CES-D scale (p=0.460).

Few participants indicated having a disability, but those who did exclusively identified as informal workers. Higher percentages of informal workers also reported having a work-related illness as a DW (6.8%, 95% CI 2.5% to 11.2%) compared with formal workers (1.%, 95% CI 0.0% to 3.0%) (p=0.129). Similarly, a higher percentage of informal workers reported having a COVID-19 infection (42.7%, 95% CI 32.1% to 53.3%) compared with formal workers (32.2%, 95% CI 9.7% to 54.6%) (p=0.250). 100% of formal workers reported having three or more doses of the COVID-19 vaccine, while 13.2% of informal workers reported having zero, one or two doses of the vaccine.

## Discussion

This study examined the health status and healthcare access among women DWs in three Peruvian cities—Lima, Piura and La Libertad—using respondent-driven sampling to reach this often-hard-to-reach population. A key focus of the study was to assess differences in health outcomes and access to healthcare services between formally and informally employed DWs.

We found that healthcare coverage varied among employment groups. Approximately 28.2% of formal workers in our sample were covered by Social Security Insurance (EsSalud), which aligns with the national average of 36% coverage. In contrast, informal workers more frequently relied on the publicly funded Integral Health Insurance (or Seguro Integral de Salud (SIS) programme in Spanish). Although SIS was expanded in 2022 to enhance access among poor and vulnerable populations, numerous deficiencies persist, including limited medication availability, staffing shortages and insufficient infrastructure.^37 38^ By contrast, EsSalud facilities typically offer more consistent medication supply, contract with third-party clinics to reduce wait times and provide broader specialist access.^39^ The ‘Domestic Workers Law’ (Law number 31047) entitles DWs to access to healthcare under EsSalud, but, given the ongoing informality of the profession, only 17.1% of our sample had access to this programme.

Patterns of healthcare utilisation revealed further challenges. Most DWs, regardless of insurance type, identified specialists—not general practitioners or family physicians—as their primary source of care. This preference aligns with broader trends in Peru, where limited confidence in primary care providers’ capacity to manage chronic or complex conditions leads patients to bypass primary care in favour of hospitals and specialists or emergency departments.^40^,^42^ Long wait times and limited consultation availability among primary care providers in both EsSalud and SIS systems further exacerbate this phenomenon.^37^

Financial barriers were also significant. Informal workers reported higher out-of-pocket health expenditures than their formally employed counterparts. This may reflect both the exclusion from formal insurance schemes and a tendency to avoid public facilities due to perceptions of overcrowding and poor quality. Consequently, many informal workers seek care in private clinics or directly from pharmacies, where services are paid for entirely out of pocket. Interestingly, in our sample, a higher percentage of informal workers (14.4%) accessed private healthcare facilities than formal workers (4.7%). In addition, in our sample, 6.8% of all DWs and 7.7% of informal workers reported using pharmacies as their primary source of care. These findings are consistent with prior research indicating that informal workers in Peru bear a higher financial burden for healthcare, often paying for transportation, medications and informal fees, in addition to losing income due to missed work.^43^

Moreover, access to care may be hindered by employment-related barriers. In our study, more informal workers reported difficulties obtaining their employers’ permission to attend medical appointments. This aligns with international literature highlighting that limited labour protections for DWs often translate into constrained access to healthcare services, with increased reliance on out-of-pocket spending and heightened financial strain.^44^,^46^

Our findings indicate a disproportionately high prevalence of obesity among DWs, with 43.0% meeting the criteria for obesity, compared with 28.2% among the general population of Peruvian adult women.^4^ Similarly, the prevalence of depressive symptoms, as measured by the CES-D scale, was markedly higher among DWs (41.4%) than the national average of 6.97% reported by the 2022 Demographic and Family Health Survey.^47^ Notably, the proportion of participants screening positive for depression (41.4%) substantially exceeded the proportion who self-reported a formal diagnosis (8.8%), possibly suggesting gaps in both mental health service utilisation and diagnostic coverage. Nationally, access to mental health services in Peru remains limited, with most of the care still concentrated in psychiatric hospitals located in Lima.^48^ While the National Mental Health Strategy has sought to integrate mental health services into primary care settings, implementation gaps persist.^49^ These include underdiagnosis, inadequate follow-up and widespread social stigma, all of which discourage individuals—especially those in precarious employment—from seeking care.^49^

Our study also explored COVID-19 vaccination coverage and found formal workers were often fully vaccinated against COVID-19. This may be attributed to employment-based requirements, under which employers were obligated to verify workers’ vaccination status before allowing them entry into the household. In cases where workers were unvaccinated, their contracts could be suspended without pay, creating a strong incentive for formal workers to become fully vaccinated.^50^ In contrast, informal workers, who were not subject to the same contractual obligations, may have had less consistent vaccination uptake. This finding highlights the role of employment conditions not only in shaping access to healthcare but also in influencing public health outcomes during health emergencies.

Our findings are consistent with other research across Latin America, including studies conducted in Ecuador, Brazil, Uruguay and Mexico. It underscores that domestic work frequently occurs in informal and unstable settings and disproportionately impacts women.^51^,^53^ Previous research from Brazil also highlights that DWs in the region were more likely to experience depression and anxiety than women employed in other sectors.^53 54^

Our findings underscore the intersectionality of employment informality, healthcare access and health inequities among DWs in Peru. Despite recent policy initiatives, substantial gaps remain in addressing the healthcare needs of this vulnerable population, necessitating more inclusive social protection mechanisms to support improved health and access to healthcare.

### Limitations

This study has several limitations. First, this study focuses on the experiences of DWs in three Peruvian cities. These locations were selected because they reported the highest numbers of DWs according to the national household survey. Nevertheless, we acknowledge that these findings may not capture the experiences of DWs in other regions. Second, while RDS is well-suited for reaching hidden and marginalised populations, it may not yield a fully representative sample of all DWs in Peru, particularly those in rural areas or without strong social networks. Third, all data were self-reported, which may introduce potential recall and social desirability biases, especially regarding sensitive topics such as mental health diagnoses, employment status or experiences of workplace barriers. Fourth, the prevalence of health conditions may be underestimated due to limited access to healthcare and diagnostic services. Fifth, we compared women DWs in both formal and informal employment; however, our formal employment group included only 49 participants, which may have affected the analysis. Because of this limited sample size, many findings were interesting and suggested possible trends but did not reach statistical significance. This outcome highlights the scarcity of formal workers in the population. Our team used innovative RDS methods to recruit as many formal workers as possible despite limited resources. Sixth, this study was not originally powered or designed to detect differences in healthcare access or health status between formally and informally employed DWs based on the sample size plan. Finally, for certain stratified comparisons, small sample sizes resulted in non-estimable RDS-II CIs.

## Conclusions

This study offers critical insights into the health status and healthcare access of women DWs in Peru, revealing disparities associated with employment informality. Despite high enrolment in public health insurance, we found that a higher proportion of informal workers experienced barriers to accessing healthcare, higher out-of-pocket expenses and higher burden of mental and physical health issues when compared with formal workers. These findings highlight persistent gaps in the implementation of labour and health protections for DWs. Addressing these inequities requires targeted policy reforms, improved enforcement of existing labour laws and investment in accessible, quality healthcare. Strengthening protections for this essential yet marginalised workforce is vital for advancing gender, labour and health equity in Peru.

## Supplementary material

10.1136/bmjph-2025-004199 online supplemental file 1

10.1136/bmjph-2025-004199 online supplemental file 2

10.1136/bmjph-2025-004199 online supplemental file 3

10.1136/bmjph-2025-004199 online supplemental file 4

## Data Availability

Data are available on reasonable request.
