# Supplementary material for: Characterising access to healthcare and the health status of women domestic workers in Peru: a respondent-driven sampling study
Source: BMJ Public Health. 2026 Feb 5;4(1):e004199. doi: 10.1136/bmjph-2025-004199 (PMC12878187; doi:10.1136/bmjph-2025-004199)
Supplement: online supplemental file 1 [file bmjph-4-1-s001.pdf]

Supplemental File 1. Participation Coupon

FICHA DE COMPENSACIÓN

Número de ficha: ______________

____

Si cuenta con ficha de compensación contáctenos al **941-493-235** previa a su visita a cualquiera de nuestras sedes en:

Lima Sur - UPCH:
Armendariz 497, Miraflores (03 cuadras de Larcomar)

Lima Norte - UPCH:
Av. Honorio Delgado 430, San Martín de Porres (cerca al Hospital Cayetano Heredia)

Encuesta para Trabajadoras del Hogar

FICHA DE PARTICIPACIÓN

Número de ficha:

CITAS: Si ha recibido una ficha de participación, llamar al 941-492-235 a partir de las 10:00am hasta las 7:00pm.

La encuesta se aplicará en la sede de estudio que prefiera:

Sedes de Estudio:

Lima SUR: Local UPCH, Arméndariz 497, Miraflores

Día y hora: Miércoles □; Jueves □; Sábado□; Domingo□; de 10:30 am hasta 6:30pm

Lima NORTE: Local UPCH, Av. Honorio Delgado 430, San Martín de Porres

Día y hora: Miércoles □; Jueves □; Sábado□; Domingo□; de 10:30 am hasta 6:30pm

Los días lunes no se aplicarán encuestas

Ficha de Compensación :

Podrá comunicar e invitar a otras compañeras para que participen de este proyecto

Recibirá una compensación por su invitación para que otras compañeras sean parte del estudio, siempre y cuando:

- **La persona invitada cumpla con los criterios de participación del estudio.**
- **La invitada no haya participado en la encuesta anteriormente.**
- **Esta ficha se entregue antes cierre del estudio (17 de diciembre 2023).**

Dudas y/o consultas:

Comunicarse con el proyecto ANITA al Whatsapp: **941-493-235**, o al correo --> cronicas.proyecto.anita@upch.pe

Ficha de participación:

¡La invitamos a participar de una investigación para que nos cuente sobre su situación actual como trabajadora del hogar!

Esta ficha le permite participar en la encuesta si cumple los sgtes. criterios de selección del estudio:

- **Identificarse como mujer.**
- **Ser trabajadora del hogar en una o más casas.**
- **Residir y trabajar en Lima**
- **Tener 14 años en adelante**

Esta ficha no se aceptará por las siguientes razones:

- **Si no cumple los criterios de selección.**
- **Si ha participado en la encuesta anteriormente.**
- **Si se entrega luego del cierre del estudio (17 de diciembre 2023)**

Dudas y/o consultas

En caso de dudas y/o consultas puede comunicarse con el proyecto ANITA al celular/Whatsapp: **941-493-235**, **a partir de las 10:00am hasta las 7:00pm** o al correo del proyecto--> cronicas.proyecto.anita@upch.pe
