# Supplementary material for: Characterising access to healthcare and the health status of women domestic workers in Peru: a respondent-driven sampling study
Source: BMJ Public Health. 2026 Feb 5;4(1):e004199. doi: 10.1136/bmjph-2025-004199 (PMC12878187; doi:10.1136/bmjph-2025-004199)
Supplement: online supplemental file 2 [file bmjph-4-1-s002.pdf]

Supplemental File 2. Survey (Spanish)

ANITA: "Abordando los Desafíos y Limitaciones de las Políticas de Protección Social para las Trabajadoras del Hogar en el Perú"

Módulo 1 - Características Sociodemográficas y Económicas

SECCIÓN 1 - EDUCACIÓN Y ETNICIDAD

Duracion ______________________________

1 ¿Cuál fue el año o grado de estudios más alto que aprobó? (Respuesta única, espontánea)

- ○ Sin estudios
- ○ Inicial
- ○ Primaria incompleta
- ○ Primaria completa
- ○ Secundaria incompleta
- ○ Secundaria completa
- ○ Superior No Universitaria
- ○ Superior Universitaria
- ○ Posgrado
- ○ No sabe/ No recuerda

2 ¿Actualmente está asistiendo a la escuela, colegio, instituto superior o universidad? (Respuesta única, espontánea)

- ○ A la primaria
- ○ A la secundaria
- ○ Al instituto superior o pedagógico
- ○ A la universidad
- ○ No asiste a ninguno

3 (Si NO respondió la opción 5 en la pregunta anterior) ¿En qué año de estudios se encuentra? (Respuesta única, espontánea)

- ○ Primero
- ○ Segundo
- ○ Tercero
- ○ Cuarto
- ○ Quinto
- ○ Sexto

4 (Si responde 2, 3, 4 o 5 en la p.1) ¿Cuál fue la principal razón por la que usted dejó de estudiar? (Respuesta única, espontánea)

- ○ Quedó embarazada
- ○ Se casó / Se unió (Unión de hecho)
- ○ Tenía que cuidar a niño/as más pequeños
- ○ Tenía que ayudar en el negocio familiar (p.ej. ayudar en la chacra)
- ○ Falta de dinero / Necesitaba trabajar
- ○ Enfermedad
- ○ No quiso estudiar
- ○ Escuela muy lejos/ No había escuela
- ○ Otro ______
- ○ No sabe

5 ¿Cuál fue el año o grado de estudios más alto de su madre? (Respuesta única, espontánea)

- ○ Sin estudios
- ○ Inicial
- ○ Primaria incompleta
- ○ Primaria completa
- ○ Secundaria incompleta
- ○ Secundaria completa
- ○ Superior No universitaria
- ○ Superior Universitaria
- ○ Posgrado
- ○ No sabe/ No recuerda

6 ¿Cuál fue el año o grado de estudios más alto de su padre? (Respuesta única, espontánea)

- ◯ Sin estudios
- ◯ Inicial
- ◯ Primaria incompleta
- ◯ Primaria completa
- ◯ Secundaria incompleta
- ◯ Secundaria completa
- ◯ Superior No universitaria
- ◯ Superior Universitaria
- ◯ Posgrado
- ◯ No sabe/ No recuerda

Ahora me gustaría que usted lea en voz alta la siguiente frase: (muestre la cartilla 1 a la entrevistada).
Nota: Ver instrucciones para esta pregunta y cartilla de lectura al final de este documento.

"Mi Perú es maravilloso. Tiene ríos, lagos, lagunas, montañas, plantas y muchos animales. Su clima es variado según sus regiones".

SOBRE PREGUNTA 7:

OBJETIVO: Conocer si la informante con nivel de instrucción primaria o menos, sabe leer o no; así como determinar la cobertura de enseñanza por la modalidad de alfabetización (sistema no escolarizado).

DILIGENCIAMIENTO DE LA PREGUNTA 7:

Utilice la cartilla de lectura para comprobar si la entrevistada sabe leer o no, y con qué facilidad lee, cuando sólo accedieron al nivel de educación primaria o menos y de acuerdo al resultado de la prueba seleccione uno de los códigos del "1" al "4" y si la persona es invidente o tiene dificultad que le impida leer (miopía, falta de lentes adecuados, etc.) pese a saber leer seleccione el código "5"

7 Si la entrevistada no puede leer toda la frase, repregunte, ¿Puede Ud. leer parte de alguna la frase?

Nota: Ver instrucciones para esta pregunta y cartilla de lectura al final de este documento.

- ○ No puede leer
- ○ Puede leer solo parte de la frase
- ○ Puede leer la frase
- ○ No hay tarjeta en el idioma requerido ______
- ○ Ciega/Problemas visuales

8 ¿Cuál es el idioma o lengua materna con el que aprendió hablar en su niñez? (Respuesta única, espontánea)

- ○ Castellano
- ○ Quechua
- ○ Aimara
- ○ Otra lengua nativa u originaria ______
- ○ Lengua extranjera ______

9 ¿Qué idioma hablan habitualmente en su hogar (donde vive con su familia)? (Respuesta única, espontánea)

- ○ Castellano
- ○ Quechua
- ○ Aimara
- ○ Otra lengua nativa u originaria ______
- ○ Lengua extranjera ______

10 ¿Qué idioma hablan habitualmente en su centro de labores principal? (Respuesta única, espontánea)

- ○ Castellano
- ○ Quechua
- ○ Aimara
- ○ Otra lengua nativa u originaria ______
- ○ Lengua extranjera ______

11 Por sus costumbres y sus antepasados, ¿Usted se siente o considera...? (Respuesta única, espontánea)

- ☐ Quechua
- ☐ Aimara
- ☐ Nativa o indígena de la Amazonía
- ☐ Perteneciente o parte de un pueblo indígena u originario
- ☐ Afroperuana o Afrodescendiente/Negra /Morena/ Zamba/Mulata
- ☐ Blanca
- ☐ Mestiza
- ☐ Asiática descendiente
- ☐ Otro ______
- ☐ No sabe/No responde

12 ¿Usted diría que profesa la siguiente religión? (Respuesta única, espontánea)

- ○ Catolicismo
- ○ Cristiana (Evangélica)
- ○ Cristiana (Protestante, Adventista)
- ○ No profeso ninguna religión
- ○ Otra religión ______

SECCIÓN 2 - LUGAR DE RESIDENCIA

13 ¿Su residencia actual es el mismo lugar donde usted trabaja?
- ◯ Sí
- ◯ No

14 ¿En qué lugar trabaja como ocupación principal? (Leer categorías)

1. Departamento: 2. Provincia:

______

______

3. Distrito: 4. Centro Poblado:

______ ______

15 ¿En qué lugar vive con su familia? (Leer categorías)

1. Departamento: 2. Provincia: 3. Distrito: 4. Centro Poblado:

______ ______ ______ ______

16 ¿Cuánto tiempo tiene usted viviendo continuamente en su residencia actual?
- ☐ Años ______
- ☐ Meses ______
- ☐ Siempre

17 Antes de que usted viniera a vivir aquí, ¿su hogar se localizaba en una ciudad, en un pueblo, o en el campo? (Respuesta única)
- ◯ Ciudad
- ◯ Pueblo
- ◯ Campo

18 ¿Nos podría especificar dónde vivía antes que usted viniera a vivir aquí? (Leer categorías)

1. País 2. Departamento: 3. Provincia: ______

______ ______

4. Distrito: 5. Centro Poblado:

______ ______

19 Entre marzo de 2020 a diciembre de 2021, ¿usted cambió de lugar de residencia?
- ◯ Sí
- ◯ No

20 ¿Nos podría especificar a dónde cambió de lugar de residencia en ese período? (Leer categorías)

1. País 2. Departamento: 3. Provincia: ______

______ ______

4. Distrito: 5. Centro Poblado:

______ ______

21 ¿Usted considera que este cambio de residencia se debió a la pandemia del por la COVID-19 (coronavirus)?
- ◯ Sí
- ◯ No

22 ¿Usted volvió a residir en el lugar donde vivía antes de la pandemia por la COVID-19?
- ◯ Sí
- ◯ No

SECCIÓN 3 - COMUNICACIONES

23 ¿Con qué frecuencia usted accede a internet (ya sea Casi todos los días desde un celular o una computadora)? (Respuesta Una vez por semana única, leer alternativas)

De vez en cuando

○
○
○ Nunca
○

24 ¿Desde qué equipo accede a internet? (Respuesta única, leer alternativas)

- ○ Desde mi celular
- ○ Desde el celular de alguien cercano
- ○ Desde una computadora en casa
- ○ Desde una computadora en el trabajo
- ○ Desde un lugar público, cabinas de internet, librería, etc.

25 En los últimos 12 meses, ¿usted ha usado un celular o alguna computadora para lo siguiente? (Leer alternativas, respuesta múltiple)

- ☐ Revisar noticias de familiares y/o conocidos
- ☐ Revisar noticias del país y el mundo
- ☐ Buscar información médica para usted
- ☐ Usar email/correo electrónico para comunicación en general
- ☐ Usar internet (email/videoconferencia) para comunicarse con un profesional de salud o centro de atención en salud
- ☐ Recibir o revisar resultados médicos
- ☐ Hacer citas con algún proveedor de atención en salud

26 Algunas veces las personas usan el internet para conectarse con otras personas a través de aplicaciones de mensajería (WhatsApp, Messenger, Telegram), además de las redes sociales (como Facebook, Twitter, Instagram, etc.). En los últimos 12 meses, ¿usted ha usado un celular o alguna computadora para lo siguiente? (Leer alternativas, respuesta múltiple)

- ☐ Para comunicarse con familiares o amistades por mensajería como WhatsApp, Messenger, etc.
- ☐ Para visitar una red social como Facebook, Twitter, etc.
- ☐ Para compartir información de salud que se difunde en redes sociales (tales como Facebook, Twitter, etc.)
- ☐ Para participar en foros o reuniones virtuales sobre salud
- ☐ Para participar en foros o reuniones virtuales sobre derechos laborales o temas relacionados a trabajadoras del hogar
- ☐ Para mirar videos en YouTube sobre temas relacionados a salud

¿Cuál es la aplicación o red social que usa con mayor frecuencia para comunicarse y/o acceder a información? ¿Y la segunda más frecuente? ¿Y la tercera más frecuente? (Espontánea, indicar número 1, 2 o 3 de acuerdo a preferencia)

	1er lugar	2do lugar	3er lugar
27.1 WhatsApp	○	○	○
27.2 Facebook	○	○	○
27.3 Instagram	○	○	○
27.4 Twitter	○	○	○
27.5 TikTok	○	○	○
27.6 YouTube	○	○	○
27.7 Otro	○	○	○

Especifique la red social que utiliza __________________________________

SECCIÓN 3 - COMUNICACIONES

¿Qué aplicación o red social usa con mayor frecuencia para comunicarse y/o acceder a información?

- ○ WhatsApp
- ○ Facebook
- ○ Instagram
- ○ Twitter
- ○ TikTok
- ○ YouTube
- ○ Otro ______

28 En su tiempo libre (fuera del trabajo) es más probable que usted vaya a un / una: (Leer alternativas, respuesta única) *Encuestador/a: En caso haya más de una alternativa de respuesta, solicitarle a la persona entrevistada que indique aquella actividad que realiza con mayor frecuencia.

- ○ Iglesia
- ○ Actividad artística (música, danza, museos, etc.)
- ○ Conversatorios o charlas informativas sobre algún tema en particular
- ○ Centro de estudios
- ○ Local partidario o sindical
- ○ Visita a su familia o amistades
- ○ Paseo sola/familia/amistades
- ○ Practica de algún deporte
- ○ Usar Internet (para navegar, comunicarse, ver videos, etc.)
- ○ Actividad económica o productiva
- ○ Descanso/ reposo / duerme
- ○ No tengo tiempo libre
- ○ Ninguno de los anteriores
- ○ Otra actividad. Especifique____________

Por favor, especifique

____

SECCIÓN 4 - CARACTERÍSTICAS DEL HOGAR

29 Actualmente, ¿usted vive sola?

○ Sí

¿Me podría indicar con cuántas personas vive normalmente en su hogar (donde vive con su familia), sin contar los que se encuentran de visita? ____________________________________

Incluida usted, ¿Cuántas personas conforman su hogar? ____________________________________

Incluida usted, ¿cuántas personas aportan económicamente a su hogar? ____________________________________

¿Cuántas personas menores de 14 años habitan en su hogar? ____________________________________

○ No

30

31

32

33

34 ¿Me podría indicar quién es el jefe/jefa de hogar en su casa? (Respuesta espontánea, múltiple)

- ☐ Yo misma
- ☐ Esposo/a, compañero/a
- ☐ Hijo/a
- ☐ Papá
- ☐ Mamá
- ☐ Tío/Tía
- ☐ Yerno/Nuera
- ☐ Abuelo/Abuela
- ☐ Suegro/Suegra
- ☐ Empleador
- ☐ Otro ______

35 Ahora, me gustaría preguntarle acerca de todas las hijas e hijos que usted ha tenido durante su vida. ¿Ha tenido alguna hija o hijo nacido vivo? (Si responde afirmativamente, indicar cuántos)

- ○ No tuvo hijos
- ○ Sí tuvo hijos nacidos vivos _______
- ○ Sí tuvo hijos fallecidos _______

36 ¿Me puede indicar sus edades actuales? (separe las edades con coma) ____________________________________

37	¿Tiene alguna hija o hijo que esté viviendo con usted actualmente? (Si responde afirmativamente, indicar cuántos)	◯ Sí ______<br>◯ No
38	¿Su casa es ... ? (Leer opciones, respuesta única)	◯ Propia (con titulo de propiedad o constancia de posesión)<br>◯ Alquilada<br>◯ Hipotecada<br>◯ Cedida<br>◯ De familiares<br>◯ Otro ______
39	¿Quién o quiénes aportan para el pago del alquiler mensual o el pago de la hipoteca? (Respuesta espontánea, múltiple)	☐ Sólo yo<br>☐ Esposo/a, compañero/a<br>☐ Hijo/a<br>☐ Yerno/nuera<br>☐ Nieto/Nieta<br>☐ Padre/Madre<br>☐ Suegro/Suegra<br>☐ Hijo adoptado/Hijo de crianza<br>☐ Otro familiar

40 ¿Cuál es el material predominante en los pisos de su vivienda? (Respuesta espontánea, múltiple)

- ○ Piso natural: Tierra / Arena
- ○ Piso rústico: Cemento sin pulir o pulido / Tablones sin pulir
- ○ Losetas / Terrazos, mayólicas, cerámicos
- ○ Parquet / Alfombra / Mármol / Porcelanato
- ○ No sabe/No contesta [NO LEER]

41 ¿Cuántas habitaciones tiene en su hogar que actualmente se usan exclusivamente para dormir? __________________________________

42 ¿De dónde proviene el agua que utiliza para cocinar y asearse actualmente? (Leer opciones, respuesta única)

- ○ Red pública con medidor propio
- ○ Red pública, con medidor compartido
- ○ Red pública sin medidor
- ○ Pozo natural
- ○ Río, vertiente, lago
- ○ Camión cisterna
- ○ Otra Fuente ______

43 ¿Cuál es el tipo de servicio higiénico que tiene en su vivienda? (Leer opciones, respuesta única)

- ○ Water / inodoro conectado a la red pública de desagüe
- ○ Letrina / silo / pozo séptico
- ○ No tiene servicio higiénico
- ○ No sabe / No responde

44 ¿De dónde proviene la energía eléctrica que utiliza actualmente? (Leer opciones, respuesta única)

- ○ Red pública con medidor propio
- ○ Red pública con medidor compartido
- ○ Red pública sin medidor
- ○ De un generador propio /comunitario
- ○ Otra fuente ______
- ○ No tiene energía eléctrica

Comentarios/observaciones del encuestador (opcional) ____

ANITA: "Abordando los Desafíos y Limitaciones de las Políticas de Protección Social para las Trabajadoras del Hogar en el Perú"

Módulo 2 - Condiciones laborales

DURACION __________________________________

Hora de inicio de la segunda ronda __________________________________

1 ¿La remuneración que usted percibe como trabajadora del hogar constituye su principal fuente de ingresos? (Leer opciones, respuesta única)

- ○ Sí es mi principal fuente de ingresos
- ○ No es mi principal fuente de ingresos
- ○ No percibo ingresos económicos como trabajadora del hogar

TERMINAR ENCUESTA Agradecer a la participante y dar por terminada la entrevista

SECCIÓN 1 - HORARIOS DE TRABAJO

2 ¿Desde hace cuánto tiempo labora en su trabajo principal actual como trabajadora del hogar, es decir, donde más horas trabaja?

Años: ______ y Meses: ______ y Días: ______

---

3 En promedio, ¿cuántas horas al día labora como trabajadora del hogar? (restando el tiempo que usa para almorzar o tomar refrigerios) Indicar número de horas y minutos (Si el encuestado tiene dificultades en calcular pedirle horas promedio del día, y ayudar a calcular)

______ ______

Escribir en texto: ______

(Registrar minutos solo cuando sea necesario)

---

4 En una semana típica de trabajo, ¿cuántos días trabaja a la semana? (Respuesta única)

○ 1 ○ 2 ○ 3 ○ 4
○ 5 ○ 6 ○ 7

---

5 En una semana típica de trabajo, ¿Qué días de la semana trabaja usted? (Espontánea y múltiple)

☐ Lunes
☐ Martes
☐ Miércoles
☐ Jueves
☐ Viernes
☐ Sábado
☐ Domingo
☐ Son días irregulares o no fijos o movibles

---

6 Según su contrato o acuerdo laboral en su centro de trabajo principal (Recoger la información de la vivienda donde laboren más horas a la semana):

¿Cuántas horas debe trabajar al día? ¿Y a la semana?

______ ______

7	¿Trabaja usted los días feriados? (Leer opciones, respuesta única)	◯ No trabajo en días feriados<br>◯ Trabajo los feriados y percibo el mismo pago que cualquier día normal<br>◯ Trabajo los feriados y percibo un doble pago por ese día
8	Durante la semana, ¿tiene usted por lo menos 24 horas de descanso continuas, es decir un día completo?	◯ Sí<br>◯ No
9	Durante su jornada de trabajo, ¿qué comidas diarias toma en su centro de labores? (Leer opciones, respuesta múltiple)	☐ Desayuno<br>☐ Almuerzo<br>☐ Lonche<br>☐ Cena<br>☐ Ninguna
10	¿Qué comidas le proporciona su empleador? (Leer opciones, respuesta múltiple)	☐ Desayuno<br>☐ Almuerzo<br>☐ Lonche<br>☐ Cena<br>☐ Ninguna
11	¿Es la misma comida que comen los miembros de la familia donde usted trabaja o no?	◯ Generalmente es la misma comida<br>◯ Generalmente es una comida diferente
	¿Usted trabajó el 30 de marzo (Día Trabajadoras y Trabajadores del Hogar) del presente año?	◯ Sí<br>◯ No

12 En promedio, ¿cuánto tiempo destina cada día para tomar sus alimentos durante su horario de trabajo?
(Especificar cantidad de horas y/o minutos diarios por cada comida, completar con ceros si es necesario)

Desayuno: ______ ______
Almuerzo: ______ ______
Lonche: ______ ______ Cena:
______ ______

13 En el último mes, ¿alguna vez tuvo menos de 12 horas libres entre el final de un día de trabajo y el inicio de la siguiente jornada de trabajo?

- Sí
- No
- No sabe/ No contesta

SECCIÓN 2 - CONDICIONES DEL LUGAR DE TRABAJO

14	Su contrato o acuerdo de trabajo como trabajadora del hogar es del tipo: (Leer opciones, respuesta única)	◯ Con residencia (cama adentro)<br>◯ Sin residencia (cama afuera)
15	¿En qué lugar pernocta/duerme en su principal centro de labores? (Leer opciones, respuesta múltiple)	◯ En una habitación independiente<br>◯ En el sillón de la sala<br>◯ En otro lugar adaptado de la vivienda (biblioteca, estudio, habitación de lavado, almacén, etc.)<br>◯ Comparte habitación con algún miembro de la familia<br>◯ Comparte habitación con otra trabajadora del hogar<br>◯ Otro ______
16	¿Tiene un baño exclusivo para usted en su centro de trabajo?	◯ Sí<br>◯ No

¿Qué tan satisfecha se siente con los siguientes aspectos del lugar donde usted pernocta/duerme ?

	Muy insatisfecha	Insatisfecha	Ni satisfecha ni insatisfecha	Satisfecha	Muy satisfecha
17.1 Habitabilidad	◯	◯	◯	◯	◯
17.2 Confortabilidad/comodidad	◯	◯	◯	◯	◯
17.3 Privacidad	◯	◯	◯	◯	◯
17.4 Tamaño	◯	◯	◯	◯	◯
17.5 Ventilación	◯	◯	◯	◯	◯
17.6 Iluminación	◯	◯	◯	◯	◯
17.7 Servicios higiénicos	◯	◯	◯	◯	◯

SECCIÓN 2 - CONDICIONES DEL LUGAR DE TRABAJO

18 Como trabajadora del hogar, ¿trabaja en una o más casas/viviendas? ______

- ○ Solamente en una casa
- ○ En más de una casa

	SECCIÓN 3 - ASEGURAMIENTO PREVISIONAL Y DE SALUD	
19	¿A qué sistema de jubilación está afiliada? (Leer opciones, respuesta única)	◯ Oficina de Normalización Previsional (ONP)<br>◯ Administradora de fondos de pensiones (AFP)<br>◯ Otro<br>◯ No está afiliada<br>◯ No sabe/No responde
20	Actualmente, ¿Le descuentan o usted aporta a algún sistema de jubilación (ONP/AFP/Otros)?	◯ Sí<br>◯ No
21	¿A qué sistema de salud está afiliada? (Leer opciones, respuesta única)	◯ Seguro Integral de Salud (SIS)<br>◯ ESSALUD<br>◯ Seguro de la policía o FFAA.<br>◯ Seguro privado / Entidades prestadoras de salud (EPS)<br>◯ Otro ______<br>◯ No está afiliada<br>◯ No sabe/No responde
22	Actualmente, ¿Te descuentan o usted aporta a algún sistema de salud?	◯ Sí<br>◯ No
23	Si es´ta afialiada a ESSALUD, ¿Es su empleador quien le paga el seguro para que acceda a este servicio?	◯ Sí<br>◯ No
24	Al margen de si está afiliada o no, cuando usted necesita atención médica, ¿a dónde acude generalmente para realizar su consulta de salud? (Múltiple, espontánea)	☐ Puesto / centro de salud MINSA<br>☐ Posta / policlínico ESSALUD<br>☐ Hospital MINSA<br>☐ Hospital del Seguro (ESSALUD)<br>☐ Hospital de las FF.AA. y/o Policía Nacional<br>☐ Hospital de la Solidaridad (SISOL)<br>☐ Consultorio médico particular<br>☐ Clínica particular<br>☐ Farmacia o botica<br>☐ En su domicilio (del paciente)<br>☐ Otro ______<br>☐ No buscó atención
25	¿Por qué no acudió a los establecimientos de salud?	☐ No tuvo dinero<br>☐ Se encuentra lejos<br>☐ Demoran mucho en atender<br>☐ No confía en los médicos<br>☐ No era grave/ no fue necesario<br>☐ Prefiere curarse con remedios caseros<br>☐ No tiene seguro<br>☐ Se auto recetó o repitió receta anterior<br>☐ Falta de tiempo<br>☐ Por el maltrato del personal de salud<br>☐ El/La empleador/a no dio permiso<br>☐ Otro ______

SECCIÓN 4 - CUMPLIMIENTO DE DERECHOS LABORALES Y BENEFICIOS SOCIALES

26 ¿Qué tipo de formalidad laboral tiene usted con su empleador? (Leer opciones, respuesta única)

- ○ Tiene un contrato de trabajo registrado en el Ministerio de Trabajo y Promoción del Empleo
- ○ Tiene un contrato de trabajo legalizado notarialmente
- ○ Tiene un contrato de trabajo simple por escrito
- ○ Tiene solamente un acuerdo verbal
- ○ Tiene un contrato con un service o agencia de empleo que subcontrata/terceriza mis servicios
- ○ No tiene ningún tipo de contrato o acuerdo

27 Sobre la remuneración por su trabajo como trabajadora del hogar, ¿cuál es la modalidad de pago?

- ○ Recibe pago en efectivo
- ○ Recibe transferencia bancaria
- ○ Ambas modalidades
- ○ Otra modalidad ______

28 Sobre la remuneración por su trabajo como trabajadora del hogar, ¿con qué frecuencia recibe su remuneración? (Leer opciones, respuesta única)

- ○ Al día
- ○ A la semana
- ○ Cada quincena
- ○ Mensualmente
- ○ De manera irregular

29 Pensando en su remuneración MENSUAL por su trabajo como trabajadora del hogar. ¿Cuánto es lo que percibe en promedio al MES? (Respuesta única,

____

espontánea)

---

30 ¿Recibió gratificaciones de acuerdo a ley por su trabajo, durante el año 2022? (Leer opciones, respuesta única)

- No, ninguna gratificación
- Sí, una gratificación al año
- Sí, dos gratificaciones al año (julio y diciembre)
- No trabajó el año 2022 como trabajadora del hogar

---

31 Respecto a sus vacaciones, usted diría que: (Leer alternativas, respuesta única)

- Ya ha tomado vacaciones el último año de servicios
- Ya cumplió un año de servicios y ha acordado con su empleador sus vacaciones para el presente año
- Este año cumple un año de servicios
- No ha tomado vacaciones el último año por desconocimiento de la ley
- No ha tomado vacaciones el último año por negativa de su empleador
- El empleador le otorgó o le otorgará vacaciones, pero no remuneradas

---

32 Si tuvo un trabajo como trabajadora del hogar anterior al actual, ¿Recibió CTS al terminar su contrato o acuerdo de trabajo?

- Sí
- No
- No sabe
- No tuvo un trabajo anterior como trabajadora del hogar

---

33 ¿Sabía usted que la CTS se deposita en los meses de mayo y noviembre?

- Sí
- No

---

34 ¿Sabe usted a cuánto asciende la CTS? (Espontánea, respuesta única)

- Un sueldo completo / Medio sueldo en cada depósito
- Otra respuesta
- No sabe/No responde

---

35 ¿Alguna vez ha necesitado un descanso médico? (Leer alternativas, respuesta única)

- Sí, y fue facilitado por el empleador
- Sí, pero no fue facilitado por el empleador
- Nunca necesitó un descanso médico

---

36 ¿Alguna vez ha necesitado atención médica durante la jornada laboral? (Leer alternativas, respuesta única)

- Sí, y pudo acudir oportunamente al médico
- Sí, pero debió esperar unos días hasta que el empleador le diera permiso para acudir al médico Sí, pero
- debió esperar al día de descanso para acudir al médico
- Sí, pero nunca acudió al médico
- Nunca necesitó atención médica

---

37 En los últimos cinco años, ¿necesitó contar con Sí, y la licencia fue otorgada licencia o permiso por estado de maternidad? (Leer Sí, pero la licencia no fue otorgada alternativas, respuesta única)

No necesitó licencia o permiso por maternidad

---

38 ¿Por cuánto tiempo le otorgaron la licencia por 90 días o más maternidad?

De 45 a 90 días

Menos de 45 días

No le otorgaron licencia por maternidad

---

39 ¿Recibió usted el subsidio por maternidad otorgado Sí por ESSALUD? No

---

40 Después de dar a luz o culminada su licencia por Retornó a su mismo trabajo maternidad, describa su retorno al puesto de trabajo: Fue despedida por sus empleadores (Leer alternativas, respuesta única)

Tuvo que renunciar

Tuvo que cambiar a otro trabajo

SECCIÓN 5 - EXPERIENCIA Y RESPONSABILIDADES LABORALES

41 ¿Cuáles de las siguientes labores o actividades realiza en su trabajo principal como trabajadora del hogar? Indique todas las que realice. (Leer alternativas, respuesta múltiple)

- ☐ Limpieza/Mantenimiento/cuidado del hogar
- ☐ Cocina/ayudante de cocina
- ☐ Lavado/planchado de ropa u otros similares
- ☐ Asistencia/cuidado de niñas/os y adolescentes
- ☐ Asistencia/cuidado de personas adultas mayores y/o personas enfermas
- ☐ Asistencia/cuidado de personas con discapacidad y/o personas dependientes del hogar
- ☐ Asistencia/cuidado de mascotas domésticas
- ☐ Jardinería
- ☐ Compras del hogar/mandados
- ☐ Otros ______

42 Además de su principal trabajo remunerado, ¿tiene otro(s) empleo(s) remunerado(s)? (Leer alternativas, respuesta única)

- ○ No tiene otro trabajo remunerado
- ○ Sí, frecuentemente
- ○ Sí, ocasionalmente

43 ¿En qué actividades se desempeña en su(s) otro(s) trabajo(s) remunerado(s)? (Leer alternativas, respuesta múltiple)

- ○ Trabajadora del hogar
- ○ Comercio
- ○ Limpieza
- ○ Cocina
- ○ Otros servicios
- ○ Otras actividades

44 ¿Cuántas horas a la semana en promedio dedica a otro(s) trabajo(s) distintos de su principal trabajo remunerado? Indicar número de horas a la semana ______

En los últimos 12 meses, ¿ha cambiado su trabajo en alguno de los siguientes aspectos? (Leer alternativas, respuesta única)

	Ha aumentado	Ha aumentado	No ha cambiado	Se ha reducido	Se ha reducido mucho
				algo	algo mucho
45.1 El número de horas que trabaja a la semana	○	○	○	○	○
45.2 Su salario o ingresos	○	○	○	○	○
45.3 Sus tareas y deberes	○	○	○	○	○

SECCIÓN 5 - EXPERIENCIA Y RESPONSABILIDADES LABORALES

46 ¿Usted laboró antes como trabajadora del hogar?

- ○ Sí
- ○ No

---

47 Tomando en cuenta su experiencia anterior como trabajadora del hogar (su centro de labores anterior al actual), ¿cuánto tiempo laboró en dicho lugar?

- ○ Un año o menos
- ○ De 1 a 2 años
- ○ De 2 a 3 años
- ○ Más de 3 años
- ○ No recuerda

---

48 Durante toda su vida, ¿cuántos años en total ha laborado como trabajadora del hogar?

Años: ______ Meses: ______

---

49 En promedio, ¿cuánto gasta diariamente en transporte para desplazarse al trabajo y viceversa para su trabajo principal? (Respuesta espontánea, única)

______ ______

---

50 En total, ¿cuánto tiempo al día dedica habitualmente a desplazarse desde casa al trabajo y vuelta a casa? (Ejm. Horas 2 y Minutos 30)

Horas: ______ y Minutos: ______

---

51 ¿Durante los últimos 12 meses (o desde que empezó a trabajar como trabajadora del hogar) ha participado de actividades de formación/capacitación para mejorar sus habilidades como trabajadora del hogar? (Respuesta múltiple)

- ☐ Formación pagada o proporcionada por empleador/a
- ☐ Formación pagada por Ud. misma
- ☐ Formación gratuita impartida por el sindicato
- ☐ Formación gratuita impartida por alguna organización del Estado
- ☐ Formación gratuita impartida por alguna organización civil
- ☐ Otro ______
- ☐ Ningún tipo de formación

---

52 En general, ¿qué curso/s de formación/capacitación usted siguió en los últimos 12 meses? (indicar cantidad de horas totales) (Respuesta espontánea, múltiple)

- ☐ Cocina / gastronomía / repostería ______
- ☐ Primeros auxilios / enfermería ______
- ☐ Jardinería / paisajismo ______
- ☐ Limpieza / mantenimiento del hogar ______
- ☐ Conducción de vehículos ______
- ☐ Otro ______ ______
- ☐ Ningún curso de formación

SECCIÓN 6 - ASPECTOS FAMILIARES Y PERSONALES

53 ¿Es usted la persona que más contribuye a los ingresos de su hogar?

- ○ Sí
- ○ No
- ○ Todos por igual

54 ¿Qué número de miembros tiene su hogar (donde vive su familia), incluyéndose usted?

____

55 ¿Cuánto es el ingreso mensual familiar aproximado? (Considerando sus ingresos como trabajadora del hogar y otros ingresos en el hogar) (Leer alternativas, respuesta única) *Encuestador/a: recuerde que esta pregunta se refiere al ingreso total entre todos los integrantes de la familia que aportan dinero en el hogar. Tome en cuenta que esta pregunta es para quienes tienen más de un aportante de ingresos [adicionales a la TdH]

______ {ingreso_m_monto}

56 En general, ¿qué tan satisfecha está con las Muy satisfecha condiciones de trabajo de su principal trabajo Satisfecha remunerado? (Leer alternativas, respuesta única)

○ Ni satisfecha ni insatisfecha
○ Insatisfecha
○ Muy insatisfecha

57 ¿Qué tan preocupada está por lo difícil que sería Nada preocupada encontrar otro trabajo en caso usted quedara Poco preocupada desempleada? (Leer alternativas, respuesta única) Más o menos preocupada

○ Algo preocupada
○ Muy preocupada

58 Suponiendo que pudiera elegir libremente sobre sus El mismo número de horas que en la actualidad horas de trabajo y teniendo en cuenta la necesidad de Una cantidad de horas diferente ______
ganarse la vida: ¿cuántas horas semanales No sabe/No contesta preferiría trabajar actualmente? (Respuesta espontánea)

59 ¿Hasta qué edad le gustaría trabajar como Lo más tarde posible / hasta que las energías lo trabajadora del hogar? (Respuesta espontánea) permitan

○ Una edad determinada ______
○ No sabe/No contesta

SECCIÓN 7 - CONDICIONES DE SEGURIDAD LABORAL

En su ocupación principal y en una jornada laboral habitual, usted, ¿con qué frecuencia ...? (Leer alternativas, respuesta única)

		Siempre	Muchas veces	Algunas veces	Casi nunca	Nunca
60.1	¿Está expuesto a un nivel de ruido que le obliga a elevar el volumen de la voz para conversar con otra persona?	○	○	○	○	○

60.2 Aplica o manipula o está en ◯ ◯ ◯ ◯ ◯contacto con sustancias químicas nocivas/tóxicas como: solventes, pesticidas (productos de jardinería), bencina, limpiadores desinfectantes (tipo ácido muriático, cloro (lejía), colorantes, pintura, entre otros

60.3 ¿Respira sustancias químicas en ◯ ◯ ◯ ◯ ◯forma de polvo, humos, aerosoles, vapores, gases? (No incluye el humo de tabaco)

60.4 Manipula o está en contacto con, ◯ ◯ ◯ ◯ ◯animales (ejemplo: paseando o tratando con perros, gatos o la mascota de su empleador)

60.5 ¿Está expuesta directamente a ◯ ◯ ◯ ◯ ◯los rayos del sol o radiación por un periodo mínimo de 1 hora diaria?

61 ¿Su empleador le proporciona implementos o equipos de Sí protección personal de acuerdo a las necesidades de No
las actividades que realiza? (por ejemplo: guantes, mascarillas, delantal, guardapolvo)

62 ¿Usa usted un uniforme de trabajo (brindado por el Sí, se lo proporcionó su empleador empleador o adquirido por cuenta propia) para realizar Sí, lo adquirió por su cuenta sus labores como trabajadora del hogar en el lugar No, porque su empleador no se lo ha proporcionado donde trabaja? (Respuesta única, leer alternativas) No, porque no es necesario utilizarlo *Encuestador/a: considere que uniforme hace referencia Me dijeron que use uniforme. Pero, no quise. a "ropa de trabajo", "vestimenta laboral"; es decir: Otro ______ prenda de uso exclusivo para la jornada laboral. Ej: mandil, guardapolvo, etc.

65 ¿Qué tipo de accidente tuvo? (Leer alternativas, respuesta múltiple)
- ◯ Golpes con algún objeto
- ◯ Caídas
- ◯ Quemaduras
- ◯ Cortes, raspones o laceraciones
- ◯ Intoxicación por manipulación o inhalación de químicos
- ◯ Otro/s ______

66 Cuando usted tuvo el accidente, ¿su empleador brindó las facilidades para su atención médica, en caso esta haya sido necesaria?
- ◯ Sí
- ◯ No
- ◯ No fue necesaria la atención médica

63 ¿Usted usa los equipos de protección personal siempre que se requiere?
- ◯ Sí
- ◯ No

64 En los últimos 12 meses, ¿ha tenido algún accidente durante sus horas de trabajo?
- ◯ No he tenido accidentes
- ◯ Sí he tenido por lo menos un accidente

SECCIÓN 8 - AUSENTISMO LABORAL Y ENFERMEDAD

67 Durante los últimos tres meses, ¿cuántos días en total ha faltado o ha tenido que dejar de trabajar debido a una baja por enfermedad o por motivos de salud? ____________________________________

68 ¿Cuántos de estos días que ha faltado (por baja) fueron causados por...

accidentes de trabajo? ______

problemas de salud relacionados o empeorados por el trabajo (excluyendo los accidentes)? ______

69 Durante los últimos tres meses, ¿cuántos días ha trabajado enferma? ____________________________________

SECCIÓN 9 - CARACTERÍSTICAS DEL EMPLEADOR		
70	En su trabajo principal como trabajadora del hogar, ¿es su actual jefe/a directo un varón o una mujer?	◯ Varón<br>◯ Mujer
71	En su trabajo principal como trabajadora del hogar, ¿tiene usted algún vínculo familiar (sanguíneo o por afinidad) con su actual jefe/a directo?	◯ Sí ______<br>◯ No
72	¿Qué personas viven en la vivienda donde usted trabaja? (indique cantidad)	☐ Personas adultas (menores de 60 años) ______<br>☐ Personas adultas mayores (mayores de 60 años) ______<br>☐ Adolescentes de 15 a 18 años ______<br>☐ Niños y adolescentes de 5 a 15 años ______<br>☐ Niños menores a 5 años ______
73	¿Qué mascotas habitan en la vivienda donde usted trabaja? (indique cantidad)	☐ Perro(s) ______<br>☐ Gatos(s) ______<br>☐ Otro ______<br>☐ Ninguna mascota

En qué medida está de acuerdo o en desacuerdo con las siguientes afirmaciones: (Leer alternativas, respuesta única)

	Totalmente de acuerdo	Parcialmente de acuerdo	Ni de acuerdo ni en desacuerdo	Parcialmente en desacuerdo	Totalmente en acuerdo desacuerdo
74.1 Su jefe/a la respeta	◯	◯	◯	◯	◯
74.2 Su jefe/a le felicita y reconoce su buen trabajo	◯	◯	◯	◯	◯
74.3 Su jefe/a sabe hacer que se trabaje en equipo	◯	◯	◯	◯	◯
74.4 Su jefe/a le da su opinión sobre su desempeño laboral	◯	◯	◯	◯	◯
74.5 Su jefe/a fomenta y apoya su formación	◯	◯	◯	◯	◯

SECCIÓN 9 - CARACTERÍSTICAS DEL EMPLEADOR

75 ¿Su empleador/a le brinda alguno de los siguientes beneficios por fuera de su remuneración mensual? (Leer alternativas, múltiple)

- ☐ Ropa
- ☐ Dinero (propinas)
- ☐ Alimentación (desayuno, almuerzo y/o cena)
- ☐ Pago por tiempos extra
- ☐ Apoyo económico en emergencias (problemas económicos, emergencias médicas, etc)
- ☐ Víveres
- ☐ Gastos educativos de sus hijos
- ☐ Otros ______
- ☐ Ninguno

---

76 ¿Su empleador/a le paga a tiempo y de acuerdo con el calendario laboral?

- ○ Sí
- ○ No

---

77 Durante los últimos 12 meses ¿ha tenido algún problema de índole estrictamente laboral en el lugar donde trabaja? (Ej. Falta de pagos, vacaciones, beneficios sociales, hostigamiento, etc.)

- ○ Sí
- ○ No

---

78 ¿Reportó alguno de estos problemas laborales a la autoridad laboral (ej. SUNAFIL)?

- ○ Sí
- ○ No

---

79 ¿Cuál fue la razón para no denunciar estos problemas laborales? (Respuesta espontánea, múltiple)

- ○ Tenía miedo de perder su trabajo
- ○ Le preocupaba que las quejas podrían dañar la relación son su empleador/a
- ○ Me preocupa que mi empleador brinde referencias negativas sobre mi trabajo
- ○ Le preocupaba que le redujeran su salario o sus horas de trabajo
- ○ Le daba miedo que su empleador/a se ponga violento/a
- ○ No sabía cómo proceder / a dónde quejarse o denunciar
- ○ Otra razón ______

Durante el último año, ¿ha sido sometida durante el transcurso de su trabajo a alguna de las siguientes situaciones? (Leer alternativas, respuesta única)

	Nunca	Casi nunca	Algunas veces	Casi siempre/	siempre
80.1 Ofensas verbales	◯	◯	◯	◯	◯
80.2 Amenazas/acoso/intimidación	◯	◯	◯	◯	◯
80.3 Comportamientos humillantes	◯	◯	◯	◯	◯
80.4 Violencia física	◯	◯	◯	◯	◯
80.5 Violencia por racismo o discriminación	◯	◯	◯	◯	◯
80.6 Acoso sexual	◯	◯	◯	◯	◯

SECCIÓN 9 - CARACTERÍSTICAS DEL EMPLEADOR

81	¿Reportó alguna de estas situaciones a alguna autoridad competente? (Ej. SUNAFIL, Policía Nacional, Centro de Emergencia Mujer, Línea 100, Ministerio Público, Poder Judicial, etc.)	○ Sí<br>○ No
82	¿Cuál fue la razón para no denunciar estas situaciones? (Respuesta espontánea, múltiple)	○ Tenía miedo de perder su trabajo<br>○ Le preocupaba que las quejas podrían dañar la relación son su empleador/a<br>○ Me preocupa que mi empleador brinde referencias negativas sobre mi trabajo<br>○ Le preocupaba que le redujeran su salario o sus horas de trabajo<br>○ Le daba miedo que su empleador/a se ponga violento/a<br>○ No sabía cómo proceder / a dónde quejarse o denunciar<br>○ Otra razón ______

Comentarios/observaciones del encuestador (opcional)

____

ANITA: "Abordando los Desafíos y Limitaciones de las Políticas de Protección Social para las Trabajadoras del Hogar en el Perú"

Módulo 3 - Condiciones de salud

SECCIÓN 1 - CONDICIONES PRE EXISTENTES DE SALUD

DURACION

____

1 ¿En general, diría que su estado de salud ACTUAL es...? (Leer alternativas, respuesta múltiple)

- ◯ Excelente
- ◯ Muy bueno
- ◯ Bueno
- ◯ Regular
- ◯ Malo

3 ¿Padece alguna enfermedad o malestar crónico?

- ◯ Sí ______
- ◯ No

2 ¿Qué enfermedades o condiciones de salud tiene usted actualmente?

- ◯ Hipertensión
- ◯ Diabetes
- ◯ Asma u otras enfermedades respiratorias crónicas
- ◯ Enfermedades de la piel
- ◯ Enfermedades osteomusculares
- ◯ Otra(s) ______
- ◯ Ninguna

¿Alguna vez en su vida un/a profesional de la salud le ha diagnosticado alguna de las siguientes condiciones médicas? (Múltiple, leer alternativas)

	Sí	No
4.1 Hipertensión	○	○
4.2 Obesidad o sobrepeso	○	○
4.3 Dispepsia o malestares gástricos	○	○
4.4 Colesterol elevado	○	○
4.5 Síndrome espalda dolorosa (con dolor irradiado)	○	○
4.6 Tuberculosis	○	○
4.7 Diabetes	○	○
4.8 Hiper o Hipotiroidismo	○	○
4.9 Anemia	○	○
4.10 Rinitis	○	○
4.11 Asma	○	○
4.12 Depresión	○	○
4.13 Enfermedades del hígado, hepatitis viral Cistitis, infección urinaria	○	○
4.14 Alcoholismo/Abuso del consumo de alcohol	○	○
4.15 Trastornos del sueño	○	○
4.16 Problemas dermatológicos	○	○
4.17 Problemas de articulaciones (rodilla, hombro, codo, mano, pie) o del sistema músculo esquelético	○	○
4.18 Cáncer de algún tipo	○	○

SECCIÓN 1 - CONDICIONES PRE EXISTENTES DE SALUD

5 ¿Alguna vez le han diagnosticado alguna enfermedad relacionada con sus labores como trabajadora del hogar?

○ Sí
○ No

6 ¿Puede especificar si alguna vez le han diagnosticado con alguna de las sgtes enfermedades relacionadas con sus labores como trabajadora del hogar? (Múltiple, leer alternativas)

- ☐ Enfermedades de la piel o mucosas por exposición a químicos o disolventes del hogar (insecticidas, lejía, ácido muriático, etc.): Dermatitis alérgica o irritante
- ☐ Enfermedades del sistema osteomuscular: Lumbagos, Hernias discales, otras lesiones musculoesqueléticas: dolor o molestias crónicos en zonas como manos y muñecas (tenosinovitis), codo (epicondilitis), y síndrome del túnel carpiano, hombros, espalda, piernas.
- ☐ Enfermedad infectocontagiosa (hepatitis, COVID-19, influenza, etc.)
- ☐ Enfermedades respiratorias (asma por agentes irritantes del trabajo)
- ☐ Estrés post-traumático
- ☐ Enfermedades causadas por agentes físicos (exposición a temperaturas extremas, deterioro de audición por ruido, etc.)
- ☐ Otras enfermedades causadas por agentes químicos (por látex, cloro, disolventes orgánicos)
- ☐ Otras enfermedades ______

---

7 Actualmente, ¿toma algún medicamento para controlar sus condiciones de salud crónicas o las relacionadas con sus labores?

- ○ Sí
- ○ No

---

8 ¿Dicho(s) medicamento(s) es(son) consumido(s) por usted por prescripción médica o se automedica?

- ○ Por prescripción médica
- ○ Se automedica

---

9 ¿Con qué frecuencia consume dicho(s) medicamento(s)? (Leer alternativas, respuesta única)

- ○ De acuerdo a la frecuencia indicada por el médico
- ○ De acuerdo a su propio criterio
- ○ Solo cuando siente algún malestar
- ○ Casi nunca/cuando lo recuerda

---

17 ¿Tiene usted alguna discapacidad permanente? (Leer alternativas, respuesta única)

- ○ Sí
- ○ No

---

18 ¿Qué tipo de discapacidad? (Leer alternativas, respuesta única)

- ○ Discapacidad Física o Motora
- ○ Discapacidad Sensorial
- ○ Discapacidad intelectual
- ○ Discapacidad mental o psíquica
- ○ Discapacidad múltiple

---

19 ¿Se encuentra usted afiliada al CONADIS?

- ○ Sí
- ○ No

SECCIÓN 2 - EVENTOS RECIENTES DE SALUD

20 A continuación, formularemos cinco preguntas, cuyas
○ No tiene problemas para caminar?
respuestas deben describir mejor su salud EN EL DÍA ○ Tiene problemas leves para caminar?
DE HOY. (Respuesta única, leer alternativas). ○ Tiene problemas moderados para caminar?
En primer lugar, quisiera preguntarle sobre la Tiene problemas graves para caminar? movilidad. ¿Diría usted que, el día de hoy...
No puede caminar?
(Respuesta única, leer alternativas)

21 Ahora quisiera preguntarle sobre el cuidado personal. No tiene problemas para lavarse o vestirse solo/a?
¿Diría usted que, el día de hoy... (Leer Tiene problemas leves para lavarse o vestirse alternativas, respuesta única) solo/a?
Tiene problemas moderados para lavarse o vestirse solo/a?
Tiene problemas graves para lavarse o vestirse solo/a?
○ No puede lavarse o vestirse solo/a?

22 Ahora quisiera preguntarle sobre las actividades que No tiene problemas para realizar sus actividades realiza todos los días, por ejemplo trabajar, de todos los días?
estudiar, hacer las tareas domésticas, actividades Tiene problemas leves para realizar sus familiares o actividades de ocio. ¿Diría usted que, actividades de todos los días?
el día de hoy... (Leer alternativas, respuesta Tiene problemas moderados para realizar sus única) actividades de todos los días?
○ Tiene problemas graves para realizar sus actividades de todos los días?
○ No puede realizar sus actividades de todos los días?

23 Ahora quisiera preguntarle sobre el dolor o malestar No tiene dolor ni malestar?
físico. ¿Diría usted que, el día de hoy... (Leer Tiene dolor o malestar leve? alternativas, respuesta única) Tiene dolor o malestar moderado?
Tiene dolor o malestar intenso?
Tiene dolor o malestar extremo?

24 Por último, quisiera preguntarle sobre la ansiedad o No está ansioso/a ni deprimido/a?
depresión. ¿Diría usted que, el día de hoy... Está levemente ansioso/a o deprimido/a?
(Leer alternativas, respuesta única)
○ Está moderadamente ansioso/a o deprimido/a?
○ Está muy ansioso/a o deprimido/a?
Está extremadamente ansioso/a o deprimido/a?

En los últimos 12 meses ¿Ha tenido alguno de los siguientes eventos de salud? (Respuesta única)

	Sí	No	No recuerda	No sabe / No responde
25.1 Gripe o resfrió, nariz tapada, mocos, estornudos, fiebre, estornudos, tos, acompañados de malestar general	○	○	○	○
25.2 Diarrea (mayor número de deposiciones al día con respecto a lo habitual), náuseas, vómitos, cólicos, inapetencia, fiebre	○	○	○	○

25.3 Herida, sarpullido, hinchazón, ◯ ◯ ◯ ◯ enrojecimiento, dolor, pus o picazón en la piel que haya requerido tratamiento

25.4 Alguna infección urinaria que ◯ ◯ ◯ ◯ haya requerido tratamiento (caracterizada con ganas frecuentes de orinar, necesidad intensa y persistente de orinar, sensación de ardor u hormigueo mientras orina o inmediatamente después de orinar

25.5 Infección o inflamación de las ◯ ◯ ◯ ◯ encías o absceso dental que haya requerido tratamiento

SECCIÓN 2 - EVENTOS RECIENTES DE SALUD

26 ¿Alguna vez tuvo síntomas de COVID-19?
- ○ Sí
- ○ No

27 ¿Llegó a confirmar que tenía COVID-19 con algún tipo de prueba de laboratorio (ej. Serológica, PCR, prueba rápida)?
- ○ Sí
- ○ No

28 ¿Por qué motivo no se sometió a una prueba de descarte de COVID-19? (Respuesta espontánea, única)
- ○ No lo consideró necesario porque inició tratamiento en casa
- ○ No tuvo tiempo para hacer pruebas de descarte, por motivos laborales
- ○ No se sentía físicamente bien para acudir a un establecimiento de salud
- ○ Tuvo miedo de contagiarme establecimiento de salud
- ○ No confía en las pruebas de descarte
- ○ No confía en los establecimientos de salud
- ○ No le dieron permiso en su centro de labores
- ○ Tenía miedo de salir positivo
- ○ Porque tenía que pagar la prueba y no tenía recursos
- ○ Porque su empleador/a se lo impidió
- ○ Otro ______

29 Si enfermó/tuvo síntomas de COVID-19, ¿cómo fue su atención? (Respuesta espontánea, única)
- ○ Ninguna, sólo en casa
- ○ Atención domiciliaria con evaluación de profesional de salud
- ○ Fue atendida en emergencias
- ○ Fue hospitalizada
- ○ Necesitó ser hospitalizada, pero no había disponibilidad

30 ¿Recibió vacunación contra COVID-19?
- ○ Sí, 1 dosis
- ○ Sí, 2 dosis
- ○ Sí, 3 dosis
- ○ Sí, 4 dosis
- ○ Sí, 5 dosis
- ○ No, no recibí ninguna dosis

31 ¿Participó de algún ensayo clínico para la vacuna del COVID-19?
- ○ Sí
- ○ No

32 Durante la pandemia del COVID-19 (coronavirus), ¿usted laboró?
- ○ Sí
- ○ No

33 Si usted laboró durante la pandemia del COVID-19 (coronavirus), dígame ¿en qué laboró? (Leer alternativas, respuesta única)
- ○ Laboró desde casa
- ○ Laboró como trabajadora del hogar cama adentro
- ○ Laboró como trabajadora del hogar cama afuera
- ○ Laboró en otras actividades, fuera de casa
- ○ Otro ______

34 Si usted no laboró durante la pandemia del COVID-19 (coronavirus), dígame ¿qué actividades realizó? (Leer alternativas, respuesta única)
- ○ Ama de casa
- ○ Enfermó de COVID 19
- ○ Cuidó a alguien en su casa (especificar: niño, persona mayor de edad/anciana)
- ○ Cuidó a algún familiar enfermo por COVID-19
- ○ Otro ______

35	¿Por qué no trabajó? (Espontánea)	◯ Porque prefirió guardar la cuarentena voluntariamente de manera estricta<br>◯ Porque estuvo enferma o estaba bajo cuarentena<br>◯ Porque alguien en su hogar estaba enfermo/a o estaba bajo cuarentena<br>◯ Porque la despidieron de su trabajo<br>◯ Otro ______
36	En una escala de 1-5 ¿Qué tanto ha alterado o alteró la pandemia de COVID-19 su vida cotidiana? (Leer alternativas, respuesta única)	◯ No la ha alterado nada<br>◯ La ha alterado un poco<br>◯ La ha alterado moderadamente<br>◯ La ha alterado mucho<br>◯ La ha alterado de forma extrema<br>◯ Prefiero no responder

De acuerdo a la siguiente tarjeta, ¿por cuál de las siguientes experiencias ha pasado durante la pandemia del COVID-19? (Indique SI o NO a cada una de las siguientes situaciones) (Mostrar tarjeta 2 y leer todas las opciones)

	Sí	No	No sabe / No responde
37.1 Fui diagnosticada con COVID-19	○	○	○
37.2 Tuve miedo de contagiarme con COVID-19	○	○	○
37.3 Tuve miedo de contagiar a otros con COVID-19	○	○	○
37.4 Estuve preocupada por amigos, familia, compañeros/as, etc.	○	○	○
37.5 Sufrí estigma o discriminación de otras personas (es decir, que la gente lo trate diferente por su identidad, por tener síntomas, u otros factores relacionados con COVID-19)	○	○	○
37.6 Tuve pérdida financiera personal (por ejemplo, pérdida de su sueldo, pérdida de inversiones/retiro, cancelaciones relacionadas a viajes, pérdida de ahorro)	○	○	○
37.7 Sentí frustración o aburrimiento	○	○	○
37.8 Me quedé sin provisiones básicas (es decir, comida, agua, medicamentos, un lugar donde quedarse)	○	○	○
37.9 Tuve más ansiedad	○	○	○
37.10Tuve más depresión	○	○	○
37.11Sufrí cambios en mi rutina normal de dormir (dormir más, dormir menos)	○	○	○
37.12Hice mayor uso de bebidas alcohólicas o sustancias ilícitas	○	○	○
37.13Sentí soledad	○	○	○
37.14Sentí confusión sobre el COVID-19, sobre cómo prevenirlo, o por qué la distancia social, el aislamiento y la cuarentena eran necesarios	○	○	○
37.15 Tuve la sensación que estaba contribuyendo al bien común previniendo que yo u otros nos enfermemos con COVID-19	○	○	○
37.16Recibí apoyo emocional o social de la familia, amigos, compañeros, consejero/a, u otra persona	○	○	○
37.17Recibí apoyo financiero de la familia, amigos, compañeros, una organización u otra persona	○	○	○
37.18Tuve más dificultades o retos de lo habitual	○	○	○

SECCIÓN 3 - SALUD MENTAL

fecha_14d

____

38 A continuación, le leeré una lista de formas o maneras en las que usted podría haberse sentido durante la última semana. Por favor indique con qué frecuencia Ud. se ha sentido así en la última semana. Puede elegir entre: Casi Nunca o Nunca (menos de 1 día), Muy pocas veces (1-2 días), Ocasionalmente o algunas veces (3-4 días), Casi siempre / Siempre (5-7 días).
Yo le voy a leer las siguientes frases como si yo fuera Ud., y usted contesta.

Encuestador/a: *Recuerde a la persona entrevistada que las preguntas las responde en función de la ULTIMA SEMANA.

	Casi Nunca o Nunca (menos de 1 día)	Muy pocas veces (1-2 días)	Ocasionalmente o algunas veces (3-4 días)	Casi siempre/siempre (5-7 días)
38.1 Me han molestado cosas que normalmente no me molestan.	○	○	○	○
38.2 Tuve problemas en concentrarme con lo que hacía	○	○	○	○
38.3 Me sentí deprimida	○	○	○	○
38.4 Sentí que todo lo que hacía era un esfuerzo	○	○	○	○
38.5 Me sentí esperanzada acerca del futuro	○	○	○	○
38.6 Me sentí con miedo	○	○	○	○
38.7 No dormí bien	○	○	○	○
38.8 Me sentí feliz	○	○	○	○
38.9 Me sentí sola	○	○	○	○
39.0 No tuve ánimo	○	○	○	○

CESD-10 score

____

SECCIÓN 3 - SALUD MENTAL

39 Durante el período de cuarentena por la pandemia delSí COVID-19, ¿usted sufrió algún tipo de violenciaNo (psicológica, física o sexual) en su hogar, es decir, en la casa donde usted vive con su familia?

○
○

40 Y pensando ahora en los últimos 12 meses, ¿usted sufrió algún tipo de violencia (psicológica, física, o sexual) en su hogar, es decir, en la casa donde usted vive con su familia?

○ Sí
○ No

41 ¿Buscó ayuda en instituciones públicas por ser víctima de violencia familiar?

○ Sí
○ No

42 Durante los últimos 30 días, ¿cuántos días tomó por lo menos un trago de cualquier bebida alcohólica? (cerveza, vino, chicha, pisco, ron, etc.)

____
((números enteros))

43 Durante los últimos 30 días, en los días en que bebió alcohol, ¿aproximadamente cuántos tragos tomó en promedio?

____
((números enteros o decimales))

44 En los últimos 30 días, ¿cuántas veces consumió 4 o más bebidas alcohólicas en una sola ocasión?

____

45 ¿Con qué frecuencia fuma usted cigarrillos ahora?

- Todos los días
- Algunos días
- Muy ocasionalmente
- Nunca

SECCIÓN 4 - SALUD PREVENTIVA

46 En una semana típica, ¿cuántos días realiza alguna actividad física o hace ejercicio de al menos moderada intensidad, tales como caminar de prisa, montar en bicicleta y nadar a ritmo normal? (no incluya levantamiento de pesas)?

- ○ Nunca
- ○ 1 día por semana
- ○ 2 días por semana
- ○ 3 días por semana
- ○ 4 días por semana
- ○ 5 días por semana
- ○ 6 días por semana
- ○ 7 días por semana

---

47 En los días en los que usted hace algún tipo de actividad o ejercicio físico de intensidad por lo menos moderada, ¿por cuánto tiempo suele hacer estas actividades? Indique la cantidad de minutos al día en promedio.

Indicar minutos por día:

____

---

48 Durante los últimos 7 días, ¿en un día típico cuánto tiempo pasó sentado en casa o en el trabajo? Incluido el tiempo que pasa sentado en un escritorio, visitando amigos, leyendo, conduciendo o viajando en un automóvil, o sentado o acostado viendo televisión. Indique la cantidad de horas al día en promedio.

Indicar horas por día:

____

---

49 Considerando que las mujeres adultas deben acudir de Hace un año o menos forma periódica a chequeos preventivos de
carácter Más de 1 año hasta 2 años ginecológico, puede indicarme ¿cuándo realizó su Más de 2 hasta 3 años última
mamografía para detectar el cáncer de mama? Más de 3 hasta 5 años
(Leer alternativas, respuesta única)

○ ○ ○ ○ Más de 5 años ○ ○ Nunca me han hecho una mamografía

---

50 ¿Hace cuánto tiempo se hizo la prueba de Hace un año o menos Papanicolaou más reciente para detectar el cáncer
Más de 1 año hasta 2 años cervical? (Leer alternativas, respuesta única) Más de 2 hasta 3 años

○ ○ ○ Más de 3 hasta 5 años
○ Más de 5 años
○ Nunca me han hecho una prueba de Papanicolau
○

Comentarios/observaciones del encuestador (opcional)

____

ANITA: "Abordando los Desafíos y Limitaciones de las Políticas de Protección Social para las Trabajadoras del Hogar en el Perú"

Módulo 4 - Acceso a la salud

SECCIÓN 1 - ATENCIÓN GENERAL EN CONSULTORIO, EMERGENCIAS Y HOSPITALIZACIÓN

DURACION

____

1 A continuación realizaremos algunas preguntas sobre la atención médica que usted tuvo en los últimos doce meses. Tomando en cuenta solamente la atención ambulatoria, es decir, CONSULTAS MÉDICAS.
¿En qué tipo de establecimiento de salud atendió sus consultas médicas? (Respuesta espontánea, múltiple)

- ☐ Puesto o centro de salud MINSA
- ☐ Centro o policlínico ESSALUD
- ☐ Hospital MINSA
- ☐ Hospital ESSALUD
- ☐ Hospital de las FF.AA. y/o Policía Nacional
- ☐ Hospital de Solidaridad (SISOL)
- ☐ Consultorio médico particular
- ☐ Clínica particular
- ☐ Farmacia o botica
- ☐ Consultorio/servicio de salud de municipalidad
- ☐ Consultorio/servicio de salud de ONG o de alguna organización religiosa
- ☐ En su domicilio (del paciente)
- ☐ Otros ______

2 En los últimos 12 meses, sin contar las veces que fue a emergencia, ¿cuántas veces fue al médico, enfermera u otro profesional de la salud para atender sus consultas médicas, es decir, consultorio (diferente a una emergencia)?

- ○ Ninguna
- ○ 1 vez
- ○ 2 veces
- ○ 3 veces
- ○ 4 veces
- ○ 5-9 veces
- ○ 10 o más veces

3 Si durante los últimos 12 meses acudió alguna vez a un establecimiento de salud para atender sus consultas médicas, es decir, consultorio (diferente a una emergencia), ¿recuerda cuánto gastó en total la última vez? (incluyendo medicinas y exámenes)

- ○ Nada, todo fue cubierto por mi seguro
- ○ Menos de 20 soles
- ○ Entre 20 y 50 soles
- ○ Entre 50 y 100 soles
- ○ Más de 100 soles

monto

____

4 Ahora, realizaremos algunas preguntas sobre la Ninguna atención médica que usted tuvo en los últimos doce 1 vez meses, tomando en cuenta solamente la atención EN 2 veces
EMERGENCIAS. 3 veces

Pensando en las veces que tuvo que acudir a 4 veces Emergencias, en los últimos 12 meses, ¿cuántas 5-9 veces veces fue a emergencias de algún establecimiento de 10 o más veces salud para atenderse?

5 ¿En qué establecimientos de salud se atendió por emergencia? (Respuesta espontánea, múltiple)

- ☐ Puesto o centro de salud MINSA
- ☐ Centro o policlínico ESSALUD
- ☐ Hospital MINSA
- ☐ Hospital ESSALUD
- ☐ Hospital de las FF.AA. y/o Policía Nacional
- ☐ Hospital Solidaridad (SISOL)
- ☐ Consultorio médico particular
- ☐ Clínica particular
- ☐ Farmacia o botica
- ☐ Consultorio/servicio de salud de municipalidad
- ☐ Consultorio/servicio de salud de ONG o de alguna organización religiosa
- ☐ En su domicilio (del paciente)
- ☐ Otros ______

6 Si durante los últimos 12 meses acudió alguna vez a emergencia para atenderse, ¿recuerda cuánto gastó en total (incluyendo medicinas y exámenes) la última vez? (Respuesta espontánea)

- ○ Nada, todo fue cubierto por mi seguro
- ○ Menos de 100 soles
- ○ Entre 100 y 500 soles
- ○ Más de 500 soles
- ○ Fue cubierto por mi empleador
- ○ No recuerda

7 Ahora, realizaremos algunas preguntas sobre el servicio de HOSPITALIZACIÓN, es decir, la permanencia o internamiento de un paciente en un establecimiento de salud.
Durante los últimos 12 meses, ¿requirió usted hospitalizarse en algún establecimiento de salud?

- ○ Sí
- ○ No

8 Si respondió afirmativamente, ¿en qué establecimiento(s) de salud estuvo hospitalizada? (Respuesta múltiple)

- ☐ Puesto o centro de salud MINSA
- ☐ Centro o policlínico ESSALUD
- ☐ Hospital MINSA
- ☐ Hospital ESSALUD
- ☐ Hospital de las FF.AA. y/o Policía Nacional
- ☐ Hospital Solidaridad (SISOL)
- ☐ Clínica particular
- ☐ Consultorio/servicio de salud de municipalidad
- ☐ Consultorio/servicio de salud de ONG o de alguna organización religiosa
- ☐ Otros ______

9 ¿Cuántos días estuvo hospitalizada la última vez?

____

10 Si durante los últimos 12 meses estuvo hospitalizada Nada, todo fue cubierto por mi seguro en un establecimiento de salud, ¿recuerda cuánto Menos de 100 soles gastó en total la última vez (incluyendo medicinas y Entre 100 y 500 soles exámenes)? (Respuesta espontánea) Más de 500 soles Fue cubierto por mi empleador

11 A continuación, hablaremos sobre su estado de salud en general y la atención general en establecimientos de salud.
Durante los últimos 12 meses, ¿usted estuvo enferma?
Sí
No
No recuerda, no está seguro

---

12 ¿Buscó atención en un establecimiento de salud?
○ Sí
○ No
○ No recuerda, no está seguro

---

13 ¿Consiguió ser atendida en el establecimiento de salud al que acudió?
○ Sí
○ No
○ No recuerda, no está segura

---

14 ¿Por qué razones no consiguió atención en salud en esas oportunidades? (Leer opciones, respuesta múltiple)
○ Por falta de recursos económicos
○ Por falta de tiempo debido a las obligaciones laborales
○ Porque no contó con la autorización de su empleador para sacar/acudir a su cita
○ Porque le dieron cita en fechas muy lejanas
○ Por la demora en la atención en los establecimientos de salud
○ Por los excesivos y exagerados trámites para solicitar consulta en los centros sanitarios.
○ Por el excesivo costo de los servicios de salud
○ Por otro/s motivo/s ______

---

15 Durante los últimos 12 meses, ¿alguna vez no consiguió los medicamentos o exámenes auxiliares prescritos (análisis)?
○ Sí, una o más veces no pudo conseguir
○ No, siempre ha podido conseguir
○ No recuerda, no está segura

---

16 ¿Por qué razones no consiguió los medicamentos o exámenes auxiliares prescritos (análisis) en esas oportunidades? (Leer opciones, respuesta múltiple)
☐ Por falta de recursos económicos
☐ Por falta de tiempo debido a las obligaciones laborales
☐ Porque no contó con la autorización de su empleador para sacar/acudir a su cita
☐ Porque le dieron cita en fechas muy lejanas
☐ Por la demora en la atención en los establecimientos de salud
☐ Por los excesivos y exagerados trámites para solicitar consulta en los centros sanitarios.
☐ Por el excesivo costo de los servicios de salud
☐ Porque no había los medicamentos en la farmacia
☐ Porque la máquina de diagnóstico de imágenes no funcionaba
☐ Porque no estaba operando el servicio de laboratorio
☐ Por otro/s motivo/s ______

---

17 Durante los últimos 12 meses, ¿alguna vez necesitó un especialista de salud o alguna referencia a un hospital o centro de mayor nivel, pero no accedió a ello?
○ Sí, una o más veces no pudo conseguir
○ No, siempre ha podido conseguir
○ No recuerda, no está segura

18 ¿Por qué razones no consiguió la atención de un especialista de salud o alguna referencia a un hospital o centro de mayor nivel en esas oportunidades? (Leer opciones, respuesta múltiple)

- ☐ Por falta de recursos económicos
- ☐ Por falta de tiempo debido a las obligaciones laborales
- ☐ Porque no contó con la autorización de su empleador para sacar/acudir a su cita
- ☐ Porque le dieron cita en fechas muy lejanas
- ☐ Por la demora en la atención en los establecimientos de salud
- ☐ Por la excesiva burocracia de los establecimientos de salud
- ☐ Por el excesivo costo de los servicios de salud
- ☐ Porque no había la especialidad en ese establecimiento de salud
- ☐ Por otro/s motivo/s ______

---

19 En general, ¿cómo calificaría la calidad del servicio de salud que ha recibido durante los últimos 12 meses?

- ○ Excelente
- ○ Muy buena
- ○ Buena
- ○ Aceptable
- ○ Mala
- ○ Muy mala
- ○ Pésima

---

20 Sin incluir psiquiatras y otros profesionales de la salud mental, ¿hay algún médico, enfermera, u otro profesional de la salud en particular que usted visite más a menudo? ¿De qué especialidad?

- ○ Sí ______
- ○ No

SECCIÓN 2 - ATENCIÓN EN ESPECIALIDADES

21 En su familia, ¿usted es madre o cuidadora de algúnSí menor de 3 años o menos?No

A continuación le haremos algunas preguntas sobre la frecuencia en que fue atendida en

En los últimos 12 meses...

	Sí	No
22.1 ¿Ha recibido atención dental?	◯	◯
22.2 ¿Ha recibido atención oftalmológica (de la vista)	◯	◯
22.3 ¿Ha recibido atención en el servicio de salud mental?	◯	◯
22.4 ¿Ha recibido atención en el servicio de nutrición y dieta saludable?	◯	◯

algunas especialidades del sistema de salud durante el último año.

22.5 ¿Ha recibido atención ginecológica?	◯	◯
22.6 ¿ha recibido atención en cuestiones de violencia psicológica, física y sexual?	◯	◯

A continuación le haremos algunas preguntas sobre la frecuencia en que fue atendida en los servicios de planificación y pediátricos.

	Sí	No
23.1 En los últimos tres años, ¿ha recibido atención para planificación familiar?	○	○

23.2 En los últimos tres años, ¿ha ○ ○ recibido atención para obtener suplementos de hierro (por ser madre gestante o por tener niños menores de 03 años de edad)

23.3 En los últimos 12 meses, ¿ha ○ ○ recibido atención en el programa de vacunación infantil?

23.4 En los últimos 12 meses, ¿ha ○ ○ recibido atención en el servicio CRED (crecimiento y desarrollo) para niños menores de 03 años?

SECCIÓN 3 - ATENCIÓN RECIENTE EN ESTABLECIMIENTOS DE SALUD

24	Durante las últimas 4 semanas, ¿requirió atención de salud por enfermedad, malestar o síntomas?	◯ Sí<br>◯ No
25	Si durante las últimas 4 semanas buscó atención de salud por enfermedad, malestar o síntomas, indicar dónde se atendió: (Respuesta múltiple, espontánea)	☐ Puesto o centro de salud MINSA<br>☐ Centro o policlínico ESSALUD<br>☐ Hospital MINSA<br>☐ Hospital ESSALUD<br>☐ Hospital de las FF.AA. y/o Policía Nacional<br>☐ Hospital Solidaridad (SISOL)<br>☐ Consultorio médico particular<br>☐ Clínica particular<br>☐ Farmacia o botica<br>☐ Consultorio/servicio de salud de municipalidad<br>☐ Consultorio/servicio de salud de ONG o de alguna organización religiosa<br>☐ En su domicilio (del paciente)<br>☐ Otro ______<br>☐ No buscó atención
26	¿Por qué no acudió a los establecimientos de ESSALUD?	◯ No tuvo dinero<br>◯ Se encuentra lejos<br>◯ Demoran mucho en atender<br>◯ No confía en los médicos<br>◯ No era grave / no fue necesario<br>◯ Prefiere curarse con remedios caseros<br>◯ No tiene seguro<br>◯ Se auto recetó o repitió receta anterior<br>◯ Falta de tiempo<br>◯ Por el maltrato del personal de salud<br>◯ Otro ______

SECCIÓN 4 - ACCESO A LOS SERVICIOS DE SALUD

27 A continuación, nos gustaría hacerle algunas preguntas acerca de su salud, cuando usted se enferma y requiere atención o tratamiento médico. ¿Es para usted un gran problema... (Responder sí o no)

	Sí	No	No sabe / No responde
27.1 saber a dónde ir a atenderse?	◯	◯	◯
27.2 conseguir permiso en su centro de trabajo para ir a atenderse?	◯	◯	◯
27.3 conseguir dinero para el transporte, la consulta y/o el tratamiento?	◯	◯	◯
27.4 la lejanía de los servicios médicos?	◯	◯	◯
27.5 tener que conseguir transporte para trasladarse hasta el establecimiento de salud?	◯	◯	◯
27.6 acudir sola al establecimiento de salud?	◯	◯	◯
27.7 que no haya personal de salud femenino?	◯	◯	◯
27.8 que no haya algún personal de salud?	◯	◯	◯
27.9 que no haya medicamentos?	◯	◯	◯

SECCIÓN 4 - ACCESO A LOS SERVICIOS DE SALUD

28	¿Conoce lugares a dónde acudir para recibir atención sobre salud mental?	◯ Sí ______<br>◯ No
29	¿Conoce lugares a dónde acudir para recibir atención sobre nutrición y dieta saludable?	◯ Sí ______<br>◯ No
30	¿Conoce lugares a dónde acudir para recibir atención sobre problemas de violencia psicológica, física y/o sexual?	◯ Sí ______<br>◯ No
31	¿Con qué frecuencia tiene dificultades para entender la información escrita (diagnóstico, recetas) acerca de su estado de salud?	◯ Siempre<br>◯ Casi siempre<br>◯ A veces<br>◯ Casi nunca<br>◯ Nunca
32	¿Con qué frecuencia alguien le ayuda a leer materiales del hospital?	◯ Siempre<br>◯ Casi siempre<br>◯ A veces<br>◯ Casi nunca<br>◯ Nunca
33	¿Qué tan fácil es para usted llenar formularios del sistema de salud por sí misma?	◯ Muy fácil<br>◯ Fácil<br>◯ Ni fácil ni difícil<br>◯ Difícil<br>◯ Muy difícil

SECCIÓN 5 - CALIDAD EN LOS SERVICIOS DE SALUD

34 Si buscó atención de salud por síntomas de enfermedad durante las últimas 4 semanas, ¿cuánto tiempo duraron las siguientes etapas/actividades?

Tiempo de espera para obtener cita en el establecimiento de salud (Especificar días): ______
Tiempo de desplazamiento hacia y desde el establecimiento de salud (Especificar horas y minutos): ______ ______
Tiempo de espera en el establecimiento de salud (Especificar horas y minutos): ______ ______
Tiempo de atención en sí (Especificar horas y minutos): ______ ______

Las siguientes preguntas son acerca de su comunicación con todos los médicos, enfermeras u otros profesionales del cuidado de salud que usted vio durante los últimos 12 meses. Los profesionales de la salud...(Leer opciones, respuesta única)

	Siempre	Por lo general	A veces	Nunca

médicas por teléfono, brindadas por un profesional No

35.1 ¿Le dieron la oportunidad de hacer todas las preguntas que usted tenía sobre su salud?

35.2 ¿Le dieron la atención necesaria a sus emociones y sentimientos?

35.3 ¿Le involucraron en decisiones sobre su salud tanto como usted quiso? ◯

35.4 ¿Se cercioraron de que usted comprendía todo lo que tiene que hacer para cuidar su salud? ◯

35.5 ¿Le explicaron las cosas de una manera que usted pudiera comprender? ◯ ◯

35.6 ¿Le dedicaron suficiente tiempo? ◯ ◯

35.7 ¿Le ayudaron a manejar sus sentimientos de incertidumbre acerca de su salud y cuidado médico? ◯ ◯

---

36 ¿Ha recibido alguna vez atención/indicaciones de la salud?

◯
◯ Sí
◯ No sabe/No conoce sobre ese servicio

37

SECCIÓN 6 - SOPORTE SOCIAL

¿Tiene amigos o parientes con quienes pueda hablar sobre su salud?

◯ Sí ______
◯ No

Durante las últimas 4 semanas, indique con qué frecuencia se sintió de la siguiente manera: (Leer opciones, respuesta única)

	Casi siempre	La mayor parte del tiempo	Cerca de la mitad del tiempo	Ocasionalmente	Nunca
38.1 Me resultó fácil llevarme bien con otras personas	○	○	○	○	○
38.2 Me sentí sola	○	○	○	○	○
38.3 Tuve alguien con quien compartir lo que sentía	○	○	○	○	○
38.4 Me resultó fácil ponerme en contacto con otras personas	○	○	○	○	○
38.5 Sentí que era una carga para las personas	○	○	○	○	○

Comentarios/observaciones del encuestador (opcional) ____

Hora de fin de la primera ronda ________________________________

BREAK

RECESO DE 10 MIN Disponer de estos minutos según lo indicado en la capacitación

(No cerrar la encuesta y dar click en "Siguiente módulo" cuando termine el receso)

ANITA: "Abordando los Desafíos y Limitaciones de las Políticas de Protección Social para las Trabajadoras del Hogar en el Perú"

Módulo 5 - Conocimientos y percepciones sobre protección social

duracion ______________________________

1	¿Ha escuchado sobre la ley 31047 sobre trabajadoras y trabajadores del hogar?	◯ Sí<br>◯ No
2	¿A través de qué medio se enteró sobre esta ley sobre trabajadoras y trabajadores del hogar?	◯ A través de otra trabajadora del hogar<br>◯ A través de su sindicato u organización<br>◯ A través de la agencia de empleos<br>◯ A través de su empleador<br>◯ En la televisión<br>◯ En la radio<br>◯ En un periódico, semanario o revista<br>◯ En la red social Facebook<br>◯ En la red social Twitter<br>◯ En la red social TikTok<br>◯ En la red social Instagram<br>◯ En la red social WhatsApp<br>◯ De otra manera ______
3	Según usted, ¿qué derechos de las trabajadoras del hogar garantiza esta ley? (Respuesta múltiple)	☐ Derecho a mejor sueldo<br>☐ Derecho al cumplimiento de vacaciones<br>☐ Derecho a la salud<br>☐ Derecho a mejores condiciones de higiene en el trabajo<br>☐ Derecho a mejores condiciones de seguridad en el trabajo<br>☐ Derecho a un mejor y pertinente descanso<br>☐ Derecho a organizarse<br>☐ Derecho a reconocerse como una población con derechos
4	¿Usted considera que esta Ley es útil o no para la mejora de sus condiciones laborales?	◯ Sí es útil<br>◯ No es útil<br>◯ No sabe / No responde
5	Respecto al cumplimiento de la Ley, ¿usted diría que...? (Leer alternativas, respuesta única)	◯ Es una Ley que se está cumpliendo<br>◯ Es una Ley que no se está cumpliendo<br>◯ Es una Ley que se cumple a medias<br>◯ Es una ley que nunca se va a cumplir
6	¿En qué plazo cree usted que se empiece a cumplir totalmente esta Ley? (Leer alternativas, respuesta única)	◯ Es una Ley que se cumplirá en el corto plazo (un año o menos)<br>◯ Es una Ley que se cumplirá en el mediano plazo (más de un año, menos de cinco años)<br>◯ Es una Ley que se cumplirá en el largo plazo (cinco años a más)<br>◯ No sabe / No responde

SECCIÓN 1 - SOBRE LA LEY N° 31047

7 ¿Qué institución o autoridad es la principalLa Presidenta de la cumplimiento de esta ley?El Ministerio de Trabajo (Respuesta múltiple)El Congreso de la República

- [ ] República responsable para lograr el
- [ ]
- [ ]
- [ ] SUNAFIL
- [ ] Los sindicatos de trabajadoras del hogar
- [ ] Otras organizaciones de trabajadoras del hogar
- [ ] Las trabajadoras del hogar
- [ ]
- [ ]

Los empleadores de trabajadoras del hogar
Otro(s) ______

SECCIÓN 2 - SOBRE EL CONTRATO DE TRABAJO

8	¿Nos podría decir qué plazo tiene su contrato vigente? (Leer alternativas, respuesta única)	○ Seis meses o menos<br>○ Mayor a seis meses y menor a un año<br>○ Un año o más<br>○ Tiene plazo indeterminado<br>○ No tiene contrato de trabajo<br>○ No sabe / No responde
9	En caso cuente con contrato laboral, conoce si su empleador/a ha registrado su contrato en la página web del Ministerio de Trabajo y Promoción del Empleo?	○ Sí, está registrado<br>○ No, no está registrado<br>○ No sabe / no está segura
10	¿Tiene usted alguno de estos tipos de contrato? (Leer alternativas, respuesta única)	○ Contrato de suplencia<br>○ Contrato de emergencia<br>○ Contrato para servicio específico<br>○ Contrato de temporada<br>○ Contrato simple de trabajo doméstico<br>○ Otro tipo de contrato específico ______<br>○ No sabe / No responde
	¿Tiene algún cargo directivo en la organización a la que pertenece?	○ Sí ______<br>○ No

SECCIÓN 3 - SOBRE LA REMUNERACIÓN Y OTROS BENEFICIOS

11 A continuación, le formularemos algunas preguntas sobre algunas condiciones laborales importantes en su centro de labores. ¿Su empleador le descuenta por concepto de alimentación o alojamiento? (Leer alternativas, respuesta única)

- ◯ Le descuentan por concepto de alimentación
- ◯ Le descuentan por concepto de alojamiento
- ◯ Le descuentan por concepto de alimentación y alojamiento
- ◯ No le realizan ningún descuento y le brindan alimentación y/o alojamiento
- ◯ No toma alimentos ni se aloja en su centro de labores

12 Hace unos minutos me comentó que su empleador le paga mediante transferencia bancaria. ¿El pago mediante transferencia es realizado con su consentimiento o por acuerdo con su empleador?

- ◯ Le transfieren con su consentimiento o acuerdo entre ambas partes
- ◯ Le trasfieren sin su consentimiento o sin acuerdo entre ambas partes

13 ¿Su empleador le extiende una boleta de pago por sus labores como trabajadora del hogar? (Leer alternativas, respuesta única)

- ◯ Sí
- ◯ No

14 ¿Con qué frecuencia su empleador la extiende boletas de pago? (Leer alternativas, respuesta única)

- ◯ Cada quince días
- ◯ Cada mes
- ◯ Cada dos meses
- ◯ Irregularmente
- ◯ Otro ______

15 ¿A cuánto ascendieron los montos de las gratificaciones que recibió el año 2022? (Leer alternativas, respuesta única)

- ◯ A un sueldo mensual completo en julio y otro sueldo más en diciembre
- ◯ A una fracción del sueldo mensual en julio y otra fracción en diciembre
- ◯ A un sueldo mensual completo solamente en julio / solamente en diciembre
- ◯
- ◯

A una fracción del sueldo mensual solamente en julio / solamente en diciembre No recibí gratificación alguna

SECCIÓN 4 - SOBRE LA JORNADA LABORAL Y DESCANSOS

16 En sus horas libres, ¿usted realiza actividades fuera de su centro de labores o permanece en su centro de labores?

- ○ Realiza actividades fuera de su centro de labores
- ○ Realiza actividades en su centro de labores
- ○ Realiza actividades en su centro de labores y fuera de él
- ○ No tiene horas libres nunca
- ○ Otro ______

17 ¿Qué actividades realiza durante sus horas libres? (Respuesta espontánea, múltiple)

- ☐ Sale a visitar a su familia o amistades
- ☐ Sale de paseo
- ☐ Hace deporte
- ☐ Usa Internet (para navegar, comunicarse, ver videos, etc.)
- ☐ Estudia
- ☐ Realiza alguna actividad económica o productiva
- ☐ Descansa / reposa / duerme
- ☐ Otra actividad ______

18 ¿Cuántos días de vacaciones cree usted que le corresponde por un año de trabajo? (Espontánea)

____

SECCIÓN 5 - SOBRE EL VÍNCULO LABORAL

19	En los últimos 12 meses, ¿usted renunció a algún centro de labores donde se desempeñaba como trabajadora del hogar?	◯ Sí<br>◯ No
20	Al renunciar, ¿usted tuvo algunos de estos inconvenientes?	◯ Tuvo inconvenientes con el plazo de preaviso al empleador<br>◯ Tuvo inconvenientes con el pago CTS<br>◯ Tuvo inconvenientes con el pago de beneficios sociales<br>◯ No tuvo inconvenientes<br>◯ Otro ______
21	En los últimos 12 meses, ¿usted fue despedida de algún centro de labores donde se desempeñaba como trabajadora del hogar?	◯ Sí<br>◯ No
22	Al ser despedida, ¿qué motivo o razón le dio su empleador para su despido? (Leer alternativas, respuesta única)	◯ Por no cumplir adecuadamente con sus labores<br>◯ Por problemas económicos de su empleador<br>◯ Por solicitar aumento de sueldo<br>◯ Por mutuo acuerdo<br>◯ Por solicitar beneficios de ley<br>◯ No dio ningún motivo<br>◯ Otro ______
23	Al culminar su relación laboral, sea por renuncia o despido ¿usted recibió liquidación?	◯ Sí<br>◯ No
24	En los últimos 12 meses, ¿usted fue colocada laboralmente por alguna agencia de empleo?	◯ Sí<br>◯ No
25	¿La agencia de empleo le cobró algún importe por colocarla laboralmente? (Leer alternativas, respuesta única)	◯ Sí, un % menor al 30% de su primer sueldo.<br>◯ Sí, el aproximadamente el 30% de su primer sueldo.<br>◯ Sí, el aproximadamente el 50% de su primer sueldo.<br>◯ Sí, el 100% de su primer sueldo.<br>◯ Otro monto ______<br>◯ No

SECCIÓN 6 - SOBRE LA SINDICALIZACIÓN

26	¿Con qué frecuencia participa de actividades, reuniones de su organización de trabajadoras del hogar? (Espontánea)	◯ Semanal<br>◯ Quincenal<br>◯ Mensual<br>◯ Trimestral<br>◯ Anual<br>◯ Nunca

PARA EL(LA) ENTREVISTADOR(A):

La(s) asociación(es) marcada(s) en la pregunta 10 de la encuesta 0 es(son): nota: Toma esta información como referencia para preguntar por el tiempo de pertenencia en cada una de las siguientes asociaciones.

[pertenece_asoc_esp]

27 ¿Desde qué tiempo es parte de dicha organización(es)?

Organización 1 Número de meses: ______ Número de años: ______
Organización 2 Número de meses: ______ Número de años: ______
Organización 3 Número de meses: ______ Número de años: ______
Organización 4 Número de meses: ______ Número de años: ______
Organización 5 Número de meses: ______ Número de años: ______
Organización 6 Número de meses: ______ Número de años: ______
Organización 7 Número de meses: ______ Número de años: ______
Organización 8 Número de meses: ______ Número de años: ______
Organización 9 Número de meses: ______ Número de años: ______
Organización 10 Número de meses: ______ Número de años: ______

28	Si pertenece a alguna organización, su empleador/a le dificulta directa/indirectamente su participación en las reuniones de la organización?	◯ Sí<br>◯ No
29	Usted nos indicó que se encuentra afiliada a, por lo menos, una asociación, organización, sindicato o federación. Como integrante, ¿paga alguna cuota mensual/anual? Detallar	◯ Sí ______<br>◯ No<br>◯ No sabe
30	Hace un momento mencionó que ocupa un cargo directivo en su sindicato/federación, ¿goza de algún beneficio por ocupar dicho cargo? Especificar	◯ Si, licencia sindical de 30 días por año calendario.<br>◯ Si, otro ______<br>◯ Ninguno<br>◯ No sabe
31	Dado que nos indicó anteriormente que no se encuentra afiliada a ningún sindicato/federación, ¿se animaría a hacerlo?	◯ Sí<br>◯ No
32	¿Cuáles son sus razones para estar afiliada a un sindicato/federación? (Respuesta espontánea, múltiple)	◯ Por recomendación de personas cercanas<br>◯ Para gozar de beneficios sociales<br>◯ Para percibir una mejor remuneración<br>◯ Para tener estabilidad laboral<br>◯ Para relacionarme con mis colegas/pares<br>◯ Para tener respaldo legal<br>◯ Otro ______

33	¿Cuáles son sus razones para no estar afiliada a un sindicato/federación? (Respuesta espontánea, múltiple)	○ Por recomendación de personas cercanas<br>○ Porque no obtengo ningún beneficio<br>○ Porque no lo considero necesario<br>○ Porque cobran una cuota mensual/anual<br>○ Porque me generaría problemas con mi empleador<br>○ Porque no sé para qué sirve/de qué se trata<br>○ Porque no sé cómo afiliarme<br>○ Otro ______
34	¿Conoce si en su localidad hay algún sindicato?	○ Sí<br>○ No

SECCIÓN 7 - SOPORTE SOCIAL Y DISCRIMINACIÓN

A continuación, voy a leer una lista de enunciados acerca de su círculo social más cercano (afectivo, familiar, amical), a los que le pido responda de acuerdo a la siguiente escala: totalmente en desacuerdo, en desacuerdo, ni de acuerdo ni en desacuerdo, de acuerdo y totalmente de acuerdo (Leer alternativas, respuesta única)

	Totalmente en desacuerdo	En desacuerdo	Ni de acuerdo ni en desacuerdo	De acuerdo	Totalmente de acuerdo
35.1 Hay una persona en especial que está cerca cuando yo estoy en necesidad	○	○	○	○	○
35.2 Hay una persona en especial con la cual yo puedo compartir mis alegrías y mis penas (lamentos)	○	○	○	○	○
35.3 Mi familia realmente trata de ayudarme	○	○	○	○	○
35.4 Yo recibo la ayuda emocional y el apoyo que necesito de mi familia	○	○	○	○	○
35.5 Yo tengo una persona en especial la cual es verdaderamente una fuente de consuelo para mí	○	○	○	○	○
35.6 Mis amistades realmente tratan de ayudarme	○	○	○	○	○
35.7 Yo puedo contar con mis amistades cuando las cosas salen mal	○	○	○	○	○
35.8 Yo puedo hablar de mis problemas con mi familia	○	○	○	○	○
35.9 Yo tengo amistades con las cuales yo puedo compartir mis alegrías y mis penas (lamentos)	○	○	○	○	○
35.10 Hay una persona en especial en mi vida a quien le importa mis sentimientos	○	○	○	○	○
35.11 Mi familia está dispuesta a ayudarme a hacer decisiones	○	○	○	○	○
35.12 Yo puedo hablar de mis problemas con mis amistades	○	○	○	○	○

36 Hace un momento usted mencionó que está de acuerdoPareja/Conviviente con la frase de que hay
una "persona especial" en suEnamorado/a vida en determinadas situaciones. En relación con suAmigo/a respuesta a
la sección anterior, ¿qué relaciónProfesional de confianza (médico/a, consejero/a,
tiene con "esa persona especial o personas?terapeuta, pastor/a, sacerdote, profesor)
(Espontánea, múltiple)Compañera trabajadora

☐
☐
☐
☐
☐ Empleador/a
☐ Otro familiar
☐ Otros ______

En su vida diaria, ¿con qué frecuencia le ha sucedido alguna de las siguientes situaciones?

(Leer alternativas, respuesta única)

	Casi todos los días	Al menos una vez por semana	Algunas veces al año	Algunas veces al mes	Menos de una vez al año	Nunca
37.1 Es tratada con menos cortesía que otras personas	○	○	○	○	○	○
37.2 Es tratada con menos respeto que otras personas	○	○	○	○	○	○
37.3 Recibe un peor servicio que otras personas en un restaurante o en una tienda	○	○	○	○	○	○
37.4 La gente actúa como si pensaran que usted no es inteligente	○	○	○	○	○	○
37.5 La gente actúa como si le tuvieran miedo	○	○	○	○	○	○
37.6 La gente actúa como si pensaran que usted deshonesta	○	○	○	○	○	○
37.7 La gente actúa como si fueran mejores que usted	○	○	○	○	○	○
37.8 La gente le pone sobrenombres o le insultan	○	○	○	○	○	○
37.9 Es amenazada o acosada	○	○	○	○	○	○

SECCIÓN 7 - SOPORTE SOCIAL Y DISCRIMINACIÓN

38 ¿Cuál cree que es la razón principal para estas experiencias? (Respuesta espontánea)

- ◯ Su nacionalidad
- ◯ Su género
- ◯ Su raza o color de piel
- ◯ Su edad
- ◯ Su religión
- ◯ Su estatura
- ◯ Su peso
- ◯ Otro aspecto de tu apariencia física
- ◯ Su orientación sexual
- ◯ Su nivel de educación o nivel de ingreso
- ◯ Una discapacidad física
- ◯ Otro ______

39 Usted, en alguna oportunidad, ¿se ha sentido discriminada por su empleador?

- ◯ Sí
- ◯ No

40 En los últimos 12 meses, ¿en algún momento fue obligada por su empleador a usar mandiles, delantales u otro distintivo que la identifique como trabajadora del hogar en espacios o establecimientos públicos (como parques, plazas, playas, restaurantes, hoteles, locales comerciales, clubes sociales y otros similares)? *Encuestador/a: En caso sea necesario, especificar el término "mandil" como prenda que cuelga del cuello y es atada a la cintura que se usa

- ◯ Sí
- ◯ No

durante la jornada laboral

41 ¿Qué consecuencias negativas le trajo llevar obligatoriamente esa vestimenta? (Leer alternativas, respuesta múltiple)

- ◯ Discriminación en espacios o establecimientos públicos
- ◯ Violencia física
- ◯ Violencia psicológica
- ◯ Violencia sexual (incluye acoso)
- ◯ Otro(s) ______
- ◯ No ha tenido consecuencias negativas

SECCIÓN 8 - SOBRE LA SEGURIDAD SOCIAL Y SEGURIDAD PERSONAL

42 Hace algunos minutos, usted mencionó que está afiliada a un sistema previsional, o sea de pensiones. ¿Su empleador retiene el aporte correspondiente para el sistema de pensiones elegido por usted?

- ◯ Sí
- ◯ No
- ◯ No sabe / No responde

43 Usted, en su condición de persona extranjera, ¿se ha visto alguna vez afectada por agencias de empleadores de trata laboral?

- ◯ Sí
- ◯ No
- ◯ No sabe / No responde

SECCIÓN 9 - CONOCIMIENTO SOBRE PROGRAMAS DE PROTECCIÓN SOCIAL Y SU USO

44 De la siguiente lista de programas de protección o asistencia social, ¿cuáles conoce o ha escuchado mencionar alguna vez? (Múltiple, leer alternativas)

CUNAMÁS: Cuidado diurno que incluye juego y nutrición a niñas y niños menores de 3 años (zonas rurales y urbanas) y acompañamiento a familias (incluye gestantes)
JUNTOS: Programa de apoyo monetario directo a los hogares más pobres con gestantes, niñas, niños y adolescentes
QALIWARMA:- Programa de alimentación escolar (inicial y primaria en escuelas públicas)
PENSIÓN 65: Programa de apoyo a adultos mayores
Pensión del Programa CONTIGO para discapacidad severa y situación de pobreza Techo Propio
BECA 18: Becas integrales para carreras de educación superior Otros ______

45 ¿Ha hecho uso de algún programa de protección o Sí ______ asistencia social durante el último año? No

(subsidio para trabajadores del sector público), Bono 210 (bono para trabajadores formales), entre otros? *Encuestador/a: En caso sea necesario, especificar el término "mandil" como prenda que cuelga del cuello y es atada a la cintura que se usa durante la jornada laboral

Comentarios/observaciones del encuestador (opcional)

Hora de término de la segunda ronda ____

46 ¿Recibió usted algún bono estatal durante la pandemia del COVID-19, como Yanapay (bono de 350 soles a personas en pobreza y extrema pobreza), Bono 600

- Sí ______
- No
- No recuerdo

____
