# Supplementary material for: Characterising access to healthcare and the health status of women domestic workers in Peru: a respondent-driven sampling study
Source: BMJ Public Health. 2026 Feb 5;4(1):e004199. doi: 10.1136/bmjph-2025-004199 (PMC12878187; doi:10.1136/bmjph-2025-004199)
Supplement: online supplemental file 3 [file bmjph-4-1-s003.docx]

**Supplementary File 3**

**Table 2 (Full Version): DW Access to Healthcare stratified by Formal versus Informal Employment Contract Status**

|  | **Overall** | | **Formal** | | | **Informal** | | | **P Value^ϒ^** |
| --- | --- | --- | --- | --- | --- | --- | --- | --- | --- |
|  | **Unadjusted** | | **RDS-II adjusted estimate** | | | **RDS-II adjusted estimate** | | |  |
| **Characteristic** | **N** | **%** | **N** | **%** | **95 CI** | **N** | **%** | **95 CI** |  |
| ***Health Insurance*** | | | | | | | | | |
| **Type of Health Insurance** | 456 |  | 49 |  |  | 407 |  |  |  |
| Integral Health Insurance (SIS) | 331 | 72.6 | 29 | 57.7 | (35.4, 79.9) | 302 | 73.0 | (61.6, 84.4) | 0.129 |
| Social Security Insurance (EsSalud) | 78 | 17.1 | 14 | 28.2 | (6.5, 49.8) | 64 | 14.8 | (7.6, 22.0) |  |
| Private Insurance | 47 | 10.3 | 6 | 14.2 | (1.4, 27.0) | 41 | 12.2 | (1.1, 23.4) |  |
| **DW Contributes to Health Insurance** | 409 |  | 43 |  |  | 366 |  |  |  |
| Yes | 26 | 6.4 | 9 | 18.1 | (3.0, 33.3) | 17 | 5.2 | (0.0, 12.4) | 0.054 |
| **Employer Paid for EsSalud Insurance** | 76 |  | 14 |  |  | 62 |  |  |  |
| Yes | 23 | 30.3 | 10 | 64.8 | (19.0, 100.0) | 13 | 23.4 | (0.0, 48.2) | **0.005** |
| ***Facility*** | | | | | | | | | |
| **Type of Facility accessed in past 12 months** |  |  |  |  |  |  |  |  |  |
| **Public** | 456 |  | 49 |  |  | 407 |  |  |  |
| Yes | 393 | 86.2 | 46 | 93.6 | (70.8, 100.0) | 347 | 83.8 | (72.9, 94.8) | 0.149 |
| **Private** | 456 |  | 49 |  |  | 407 |  |  |  |
| Yes | 65 | 14.3 | 4 | 4.7 | (1.5, 7.8) | 61 | 14.4 | (8.3, 20.5) | 0.084 |
| **Pharmacy** | 456 |  | 49 |  |  | 407 |  |  |  |
| Yes | 31 | 6.8 | 2 | 6.0 | (0.0, 29.2) | 29 | 7.7 | (0.0, 18.5) | 0.788 |
| ***Healthcare Type*** |  |  |  |  |  |  |  |  |  |
| **Primary Healthcare Provider** | 453 |  | 49 |  |  | 404 |  |  |  |
| Yes | 161 | 35.5 | 10 | 14.4 | (2.0, 26.9) | 151 | 36.3 | (27.6, 45.0) | **0.0169** |
| **Emergency Department** | 456 |  | 49 |  |  | 407 |  |  |  |
| Yes | 121 | 26.5 | 14 | 30.2 | (13.9, 46.4) | 107 | 24.0 | (16.4, 31.5) | 0.486 |
| **Barriers to Care** | | | | | | | | | |
| **Trouble Finding Medical care** | 456 |  | 49 |  |  | 407 |  |  |  |
| Yes | 173 | 37.9 | 17 | 33.8 | (12.0, 55.6) | 156 | 36.5 | (27.2, 45.9) | 0.753 |
| **Trouble getting workplace permission for HC visit** | 453 |  | 49 |  |  | 404 |  |  |  |
| Yes | 151 | 33.3 | 13 | 21.2 | (4.3, 38.2) | 138 | 32.2 | (21.3, 43.1) | 0.187 |
| **Out-of-pocket expense of last medical visit in past 12 months** | 440 |  | 48 |  |  | 392 |  |  |  |
| Greater than 100 PEN | 99 | 22.5 | 6 | 10.5 | (0.0, 32.5) | 93 | 21.2 | (14.1,28.4) | 0.249 |
| 100 PEN or less | 143 | 32.5 | 15 | 33.0 | (15.5, 50.6) | 128 | 34.2 | (22.5,45.9) |  |
| Nothing, everything is covered by insurance | 198 | 45.0 | 27 | 56.4 | (33.6, 79.3) | 171 | 44.5 | (34.1,54.9) |  |
| **Work-Related Accidents** | | | | | | | | | |
| **Accident at work in the last 12 months** | 456 |  | 49 |  |  | 407 |  |  |  |
| Yes | 62 | 13.6 | 10 | 20.6 | (0.0, 43.0) | 52 | 11.6 | (4.1, 19.1) | 0.167 |
| **Medical services provided by the employer?** | 62 |  | 10 |  |  | 52 |  |  |  |
| Yes | 25 | 40.3 | † | 11.0 | (0.0, 36.2) | † | 46.8 | (20.2, 73.4) | 0.472 |
| No | 15 | 24.2 | † | 29.4 | (0.0, 85.9) | † | 19.8 | (3.6, 35.9) |  |
| Medical attention not needed | 22 | 35.5 | 5 | 59.7 | (17.8, 100.0) | 17 | 33.5 | (6.7, 60.2) |  |

Note: CI = confidence interval, DW = domestic worker, RDS = respondent-driven sampling.

† Data was suppressed for cells with fewer than 5 individuals to protect individual confidentiality and prevent the calculation of sensitive data through subtraction from corresponding totals

*RDS2 confidence interval not estimable --- since the point estimate of the percentage is 0% or 100%.

**^ϒ^** P Value - bootstrap contingency test p-values.
