# Supplementary material for: Characterising access to healthcare and the health status of women domestic workers in Peru: a respondent-driven sampling study
Source: BMJ Public Health. 2026 Feb 5;4(1):e004199. doi: 10.1136/bmjph-2025-004199 (PMC12878187; doi:10.1136/bmjph-2025-004199)
Supplement: online supplemental file 4 [file bmjph-4-1-s004.docx]

**Supplementary File 4**

**Table 4 (Full Version): DW Health Status stratified by Formal versus Informal Employment Contract Status**

|  | **Overall** | | **Formal** | | | **Informal** | | | **P Value^ϒ^** |
| --- | --- | --- | --- | --- | --- | --- | --- | --- | --- |
|  | **Unadjusted** | | **RDS-II adjusted estimate** | | | **RDS-II adjusted estimate** | | |  |
| **Characteristic** | **N** | **%** | **N** | **%** | **95 CI** | **N** | **%** | **95 CI** |  |
| **Perceived Health Status** | 456 |  | 49 |  |  | 407 |  |  |  |
| Excellent and Very Good | 46 | 10.1 | 4 | 8.2 | (0.0, 28.3) | 42 | 11.6 | (5.1, 18.2) | 0.578 |
| Good,Fair and Bad | 410 | 89.1 | 45 | 91.8 | (71.7, 100.0) | 365 | 88.4 | (81.8,94.9) |  |
| ***Diagnosed Health Conditions*** | | | | | | | | | |
| **Hypertension** | 452 |  | 49 |  |  | 403 |  |  |  |
| Yes | 34 | 7.5 | † | 8.9 | (1.1, 16.7) | † | 8.2 | (2.9, 13.6) | 0.916 |
| **Diabetes** | 433 |  | 49 |  |  | 406 |  |  |  |
| Yes | 22 | 4.8 | † | 4.4 | (0.0, 27.9) | † | 5.1 | (0.8, 9.4) | 0.909 |
| **Hypercholesteremia** | 454 |  | 49 |  |  | 405 |  |  |  |
| Yes | 75 | 16.5 | 12 | 27.0 | (2.9, 51.2) | 63 | 13.9 | (8.3, 19.5) | 0.106 |
| **Asthma** | 456 |  | 49 |  |  | 405 |  |  |  |
| Yes | 22 | 4.8 | † | 1.6 | (0.0, 3.3) | † | 4.3 | (1.1, 7.5) | 0.349 |
| **Back Pain** | 456 |  | 49 |  |  | 407 |  |  |  |
| Yes | 25 | 5.5 | † | 11.2 | (0.0, 35.7) | † | 5.1 | (2.3, 7.9) | 0.464 |
| **Depression** | 455 |  | 49 |  |  | 406 |  |  |  |
| Yes | 40 | 8.8 | † | 1.6 | (0.0, 4.4) | † | 9.7 | (5.1, 14.4) | 0.052 |
| **Anxiety** | 454 |  | 49 |  |  | 407 |  |  |  |
| Yes | 48 | 10.5 | † | 3.5 | (0.0, 9.1) | † | 12.3 | (6.1, 18.5) | 0.322 |
| **Sleep Disorder** | 456 |  | 49 |  |  | 407 |  |  |  |
| Yes | 24 | 5.3 | † | 10.3 | (0.0, 35.9) | † | 5.8 | (1.7, 10.0) | 0.499 |
| **BMI** | 456 |  | 49 |  |  | 407 |  |  |  |
| <25.0 | 95 | 20.8 | 11 | 24.1 | (7.4, 40.9) | 84 | 23.6 | (14.3, 32.8) | 0.954 |
| 25.0 to <30.0 | 165 | 36.2 | 16 | 38.9 | (14.3, 63.6) | 149 | 36.4 | (27.2, 45.7) |  |
| 30.0+ | 196 | 43.0 | 22 | 36.9 | (19.6, 54.3) | 174 | 40.0 | (29.3, 50.8) |  |
| ***Depression based on CES-D Scale*** | 456 |  | 49 |  |  | 407 |  |  |  |
| Yes | 189 | 41.4 | 19 | 34.3 | (12.6-55.9) | 170 | 41.7 | (31.9-51.4) | 0.460 |
| **Disability Condition** | 456 |  | 49 |  |  | 407 |  |  |  |
| Yes | 9 | 2.0 | 0 | 0 | * | 9 | 1.5 | (0.4, 2.5) | N/A |
| *Health Conditions Related to Work as a DW* | | | | | | | | | |
| **Any Illness** | 456 |  | 49 |  |  | 407 |  |  |  |
| Yes | 39 | 8.6 | † | 1.4 | (0.0, 3.0) | † | 6.8 | (2.5,11.2) | 0.129 |
| **Skin Disease** | 456 |  | 49 |  |  | 407 |  |  |  |
| Yes | 5 | 1.1 | † | 0.6 | (0.0, 1.1) | † | 0.7 | (0.3, 1.1) | 0.932 |
| **MSK Disease** | 456 |  | 49 |  |  | 407 |  |  |  |
| Yes | 29 | 6.4 | † | 1.4 | (0.0,3.0) | † | 5.3 | (1.1,9.5) | 0.253 |
| ***COVID-19*** | | | | | | | | | |
| **Covid 19 Infection** | 456 |  | 49 |  |  | 407 |  |  |  |
| No or do not remember | 257 | 56.4 | 31 | 67.8 | (45.4, 90.3) | 226 | 57.3 | (46.7,67.9) | 0.250 |
| Yes | 199 | 43.6 | 18 | 32.2 | (9.7,54.6) | 181 | 42.7 | (32.1,53.3) |  |
| **COVID 19 Vaccine** | 456 |  | 49 |  |  | 407 |  |  |  |
| 1 dose | 8 | 1.8 | 0 | 0 | * | 8 | 1.1 | (0.2, 1.9) | N/A |
| 2 doses | 40 | 8.8 | 0 | 0 | * | 40 | 10.6 | (4.1, 17.1) |  |
| 3 or more doses | 402 | 88.2 | 49 | 100 | * | 353 | 86.9 | (80.2, 93.6) |  |
| no doses | 6 | 1.3 | 0 | 0 | * | 6 | 1.5 | (0.0, 3.1) |  |

Note: CI = confidence interval, DW = domestic worker, RDS = respondent-driven sampling.

† Data was suppressed for cells with fewer than 5 individuals to protect individual confidentiality and prevent the calculation of sensitive data through subtraction from corresponding totals

*RDS2 confidence interval not estimable --- since the point estimate of the percentage is 0% or 100%.

**^ϒ^** P Value - bootstrap contingency test p-values.
